# Enhanced Ca^2+^ influx in mechanically distorted erythrocytes measured with ^19^F nuclear magnetic resonance spectroscopy

**DOI:** 10.1038/s41598-021-83044-z

**Published:** 2021-02-12

**Authors:** Philip W. Kuchel, Konstantin Romanenko, Dmitry Shishmarev, Petrik Galvosas, Charles D. Cox

**Affiliations:** 1 School of Life and Environmental Sciences, University of Sydney, Building G08, Sydney, NSW 2006 Australia; 2 John Curtin School of Medical Research, Australian National University, Canberra, ACT Australia; 3 MacDiarmid Institute for Advanced Materials and Nanotechnology, School of Chemical and Physical Sciences, Victoria University Wellington, Wellington, New Zealand; 4 Victor Chang Cardiac Research Institute, Darlinghurst, Sydney, NSW Australia; 5 St Vincent’s Clinical School, Faculty of Medicine, University of New South Wales, Sydney, NSW Australia

**Keywords:** Biophysics, Cell biology

## Abstract

We present the first direct nuclear magnetic resonance (NMR) evidence of enhanced entry of Ca^2+^ ions into human erythrocytes (red blood cells; RBCs), when these cells are mechanically distorted. For this we loaded the RBCs with the fluorinated Ca^2+^ chelator, 1,2-bis(2-amino-5-fluorophenoxy)ethane-*N,N,N′,N′*-tetraacetic acid (5FBAPTA), and recorded ^19^F NMR spectra. The RBCs were suspended in gelatin gel in a special stretching/compression apparatus. The 5FBAPTA was loaded into the cells as the tetraacetoxymethyl ester; and ^13^C NMR spectroscopy with [1,6-^13^C]d-glucose as substrate showed active glycolysis albeit at a reduced rate in cell suspensions and gels. The enhancement of Ca^2+^ influx is concluded to be via the mechanosensitive cation channel Piezo1. The increased rate of influx brought about by the activator of Piezo1, 2-[5-[[(2,6-dichlorophenyl)methyl]thio]-1,3,4-thiadiazol-2-yl]-pyrazine (Yoda1**)** supported this conclusion; while the specificity of the cation-sensing by 5FBAPTA was confirmed by using the Ca^2+^ ionophore, A23187.

## Introduction

### Ca^2+^ dependent enhanced glycolysis

By using ^13^C NMR spectroscopy it was shown that human erythrocytes (red blood cells; RBCs) that are distorted by compression or stretching in gels, display acceleration of metabolism of [1,6-^13^C]D-glucose^1^. The deformation in the gels changes the usual biconcave disc shape of RBCs into other morphologies with altered membrane curvature^2^. Rate enhancements of glycolysis up to ~ 80%, depending on the extent of distortion, occur only if Ca^2+^ is present in the suspension medium^1^. Therefore, this divalent cation is a key mediator in the metabolic response. In the latter report, it was shown that the transmembrane exchange of the K^+^ congener ^133^Cs^+^ is also enhanced by the imposed shape changes. The ^133^Cs^+^ flux enhancement was recorded in a direct way because ^133^Cs NMR spectra have two peaks (resonances) from the cation on either side of the cell membrane^1,3,4^. This posed the question whether the putative enhanced Ca^2+^ flux could be directly measured. Unfortunately, to this end, the ‘split peak effect’^5^ seen with ^133^Cs^+^ is not *directly* observable with Ca^2+^, even in the presence of a shift reagent^6^. Furthermore, the NMR-active nuclide of Ca^2+^ (^43^Ca) has very poor sensitivity to detection by NMR^7^. However, important insights into calcium flux control in cells have been made possible by using ^19^F NMR spectroscopy coupled with the ^19^F-labelled Ca^2+^-chelator 1,2-bis(2-amino-5-fluorophenoxy)ethane-*N,N,N′,N′*-tetraacetic acid (5FBAPTA)^8–11^.

Accordingly, RBCs were loaded with 5FBAPTA in gelatin gels, and we used the previously described apparatus^12^ to hold them in distorted forms. Thus, Ca^2+^ influx was measured from the surrounding medium with ^19^F NMR spectroscopy. Consequently, this is the first report of (quasi-)direct NMR measurement of Ca^2+^ influx in RBCs, via the specifically identified mechanosensitive cation channel, Piezo1.

### Ca^2+^ flux mediated by Piezo1

The dependence of Ca^2+^ influx on RBC shape using radioactive ^45^Ca^2+^ in a rheometer was established in 1981 and 1982 implying molecular interactions mediated between RBC shape and membrane cation permeability^13,14^. However, this experimental approach appears not to have evolved further and methods using molecular biology were the basis of more recent advances in understanding mammalian mechanically induced cation transport. Since then, in 2010, a leap forward in our understanding of human cellular mechano-sensitivity was made with the discovery of the mechanosensitive integral membrane proteins Piezo1 and Piezo2^15–17^.

The most recent direct observation of enhanced Ca^2+^ influx occurring in response to RBC deformation was made by loading the cells with the fluorescent Ca^2+^ indicator Fluo-4 and distorting an RBC on the tip of a patch-clamp pipette under a fluorescence microscope^18^. This work was interpreted in the light of major advances in knowledge of Piezo1 in human RBCs that has been made in studies of the rare pro-haemolytic condition, hereditary xerocytosis. Dehydration of RBCs is a key cytological feature of this disorder and it has been traced to mutation(s) in the *PIEZO1* gene^19^. The mutated Piezo1 has reduced inactivation^20^ or deactivation^21^, leading to constitutively increased Ca^2+^ influx. The elevated cytoplasmic Ca^2+^ concentration stimulates K^+^ efflux via the Gárdos channel (KCa3.1; KCNN4; Ca^2+^ activated K^+^ channel) that is followed by osmotically driven water egress via Aquaporin 1, and hence dehydration of the cells^18,19^. While Piezo1 is non-selective for cations, there is differential permeability in the order K^+^  > Cs^+^, Na^+^  > Li^+^  > Mg^2+^, Ca^2+^, with the apparent dissociation binding constant/concentration at half maximal flux for K^+^ being *K*_D_ = 32 mM^22^.

### Control scheme

The proposed mechanism of the enhancement of glycolysis on distorting RBCs is: (1) Ca^2+^ enters the RBC via Piezo1 upon membrane distortion; (2) this activates plasma membrane calcium ATPase (PMCA) that pumps Ca^2+^ back out of the cytoplasm; (3) increased ATP consumption lowers cytosolic ATP concentration; and (4) this stimulates glycolysis, because glycolytic flux is positively regulated by ATP demand, via allosteric inhibition of hexokinase by free ATP^1^.

The significance of the Ca^2+^ in RBCs from the point of view of its influx pathways, phosphorylation of proteins especially the cytoskeleton, volume regulation, and phospholipid asymmetry in the plasma membrane, has been comprehensively reviewed in^23^. And, Ca^2+^ has recently been woven into the understanding of the network of intra- and extracellular control in RBC senescence^24^.

## Results

### Quantification of distortion enhanced Ca^2+^ influx

**Figure 1:**
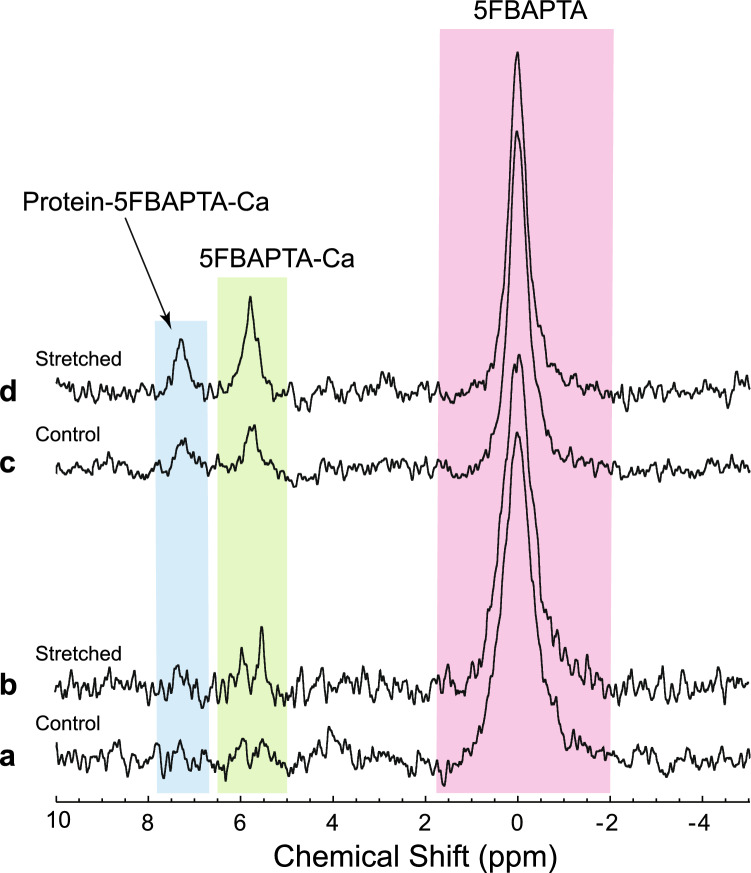
Net Ca^2+^ entry into RBCs after being stretched to 75% in gel for 42 h at 20 °C. The ^19^F NMR spectra were recorded at 37 °C after extracting the RBCs from the gel, using the protocol of Fig. S2. The pink highlighting indicates free intracellular 5FBAPTA, green, the Ca^2+^ complex of 5FBAPTA, and blue, the ternary complex between Ca^2+^ and protein inside the cells. (**a**) Control (relaxed) RBC-gel; (**b**) the stretched sample; (**c**) sample (**a**) frozen and thawed in liquid nitrogen to lyse the RBCs; (**d**) sample (**b**) treated as for (**c**) NMR settings: 5-mm ^19^F probe; 90° pulse duration was 8 µs; each FID was composed of 1024 complex data points; 128 FIDs were accumulated for each spectrum; inter-transient delay was 4.61 s; total accumulation time per spectrum was 10 min; spectral width was 10 kHz (26.71 ppm); and 20 Hz line broadening was applied by exponential multiplication of the FIDs.

We implemented a set of refinements based on the results reported below, and in the Supplementary Information (SI), to enhance the signal-to-noise ratio (S/N) in the ^19^F NMR spectra of 5FBAPTA-loaded RBCs. We reduced the possibility of artefacts brought about by hydrogen bonding of 5FBAPTA and its Ca^2+^ complex to protein inside the RBCs, and outside in the gelatin gel, indicated by the results shown in Fig. S1. Consequently, after the period of incubation of RBCs that had been stretched (or compressed) in gels the cells were removed from this medium. Details of the protocol are given in Fig. S2. In addition, the washed RBCs were haemolysed by freezing and thawing in liquid nitrogen and then the spectra were recorded not at 20 °C (as for Fig. S1) but 37 °C. The result was a much greater S/N for the 5FBAPTA peak, S/N = 31 (Fig. 1d and the control Fig. 1c), compared with 15 for the non-lysed RBCs (Fig. 1b and its control Fig. 1a), and 5 when the RBCs had been left in their gel, at 20 °C (Fig. S1). An important observation was the broad peak at ~ 7.3 ppm that was present in spectra recorded at 37 °C and not at 20 °C. To our knowledge, this has not been reported before. The interpretation of it as being due to a protein-5FBAPTA-Ca complex is given in the main Discussion, and the SI in conjunction with Figure S5.

The S/N values of the three peaks in Fig. 1d at 7.5 ppm (protein-5FBAPTA-Ca), 5.9 ppm (5FBAPTA-Ca), and 0.0 ppm (5FBAPTA) were 4.6, 8.3, and 31, respectively; and their integral ratios were 0.08:0.16:1.00. Given a total loaded concentration of 5FBAPTA of 4.0 mmol (L RBC)^−1^, the integral ratios indicated concentrations 0.26, 0.53 and 3.21 mmol [L RBC]^−1^. The total incubation time for the stretched sample was 42 h so the net Ca^2+^ flux was calculated as follows: (0.26 + 0.53) /42.0 = 18.8 µmol (L RBC)^−1^ h^−1^ at 20 °C. This is compared with the control sample values (totalling 4 mmol [L RBC]^−1^) of 0.14, 0.25 and 3.60 that implied a net Ca^2+^ influx of (0.14 + 0.25)/42.0 = 9.3 µmol (L RBC)^−1^ h^−1^ at 20 °C. The explanations of the spectral assignments are given next.

### 5FBAPTA-AM loading

Since the experiments were designed to quantify net influx of Ca^2+^, it was essential to establish that a known amount of 5FBAPTA-AM had entered the RBCs from the incubation medium. The loading procedure was refined to avoid haemolysis (~ 5%) that had occurred when the stock solution (200 mM in DMSO) was added directly to an RBC suspension.

**Figure 2:**
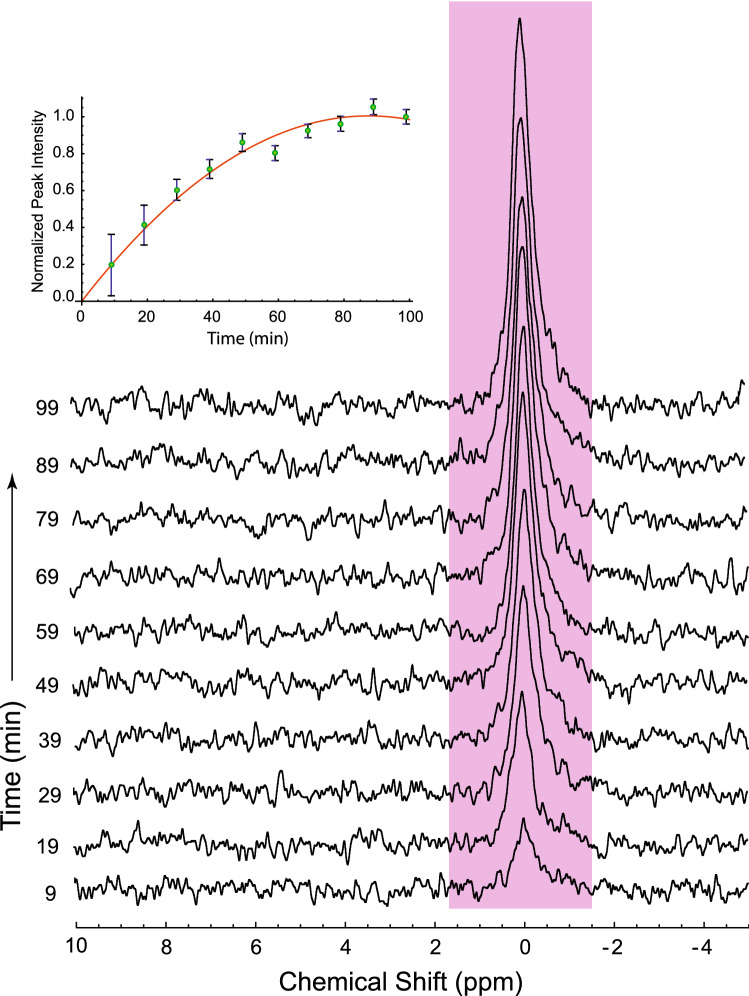
^19^F NMR spectral time course of loading 5FBAPTA into RBCs in a suspension at 37 °C. The pink area highlights the resonance of free 5FBAPTA that was assigned a chemical shift δ = 0.0. Acquisition of the first spectrum started at 4 min after mixing 10 µL of 200 mM 5FBAPTA-AM in DMSO with the 0.5 mL RBC suspension of *Ht* = 0.65. NMR settings were as for Fig. 1. The inset shows the 5FBAPTA peak intensities (green dots) from each spectrum over the 99-min time course; the time indicated is at the centre of FID accumulation. The solid red line is an empirical quadratic fit to the data [(0.023 ± 0.001) *t − *(0.00013 ± 0.00001) *t*^2^] used to obtain the initial slope at *t* = 0, and to guide the eye. The errors bars are reciprocals of the S/N derived automatically using TopSpin 4.0.

On the other hand, for the ^19^F NMR time courses showing the evolution of 5FBAPTA in RBC suspensions (not in gel), adding the 5FBAPTA-AM solution directly to the suspension was unavoidable. Figure 2 shows the emergence of the signal from free 5FBAPTA after 5FBAPTA-AM had been added to an RBC suspension with no extracellular Ca^2+^. The volume of added stock solution was calculated to give 4 mmol (L RBC)^−1^ inside the cells; this was reached in ~ 80 min as seen in the inset of Fig. 2. The concentration of 5FBAPTA was confirmed by adding a standard solution of the K^+^-salt of 5FBAPTA (K_4_5FBAPTA) to the haemolysate that was prepared from the sample, and measuring the relative peak integrals.

The initial rate of 5FBAPTA emergence was calculated as follows: The *Ht* was 0.65 and the final concentration of 5FBAPTA was 4 mmol (L RBC)^−1^, hence the normalized peak intensity in the inset of Fig. 2 had an initial rate of uptake of 0.023 × 4.0 = 0.092 ± 0.004 mmol (L RBC)^−1^ min^−1^. In other words, the apparent first order rate constant was 0.023 min^−1^.

### Yoda1 stimulated Ca^2+^ uptake

**Figure 3:**
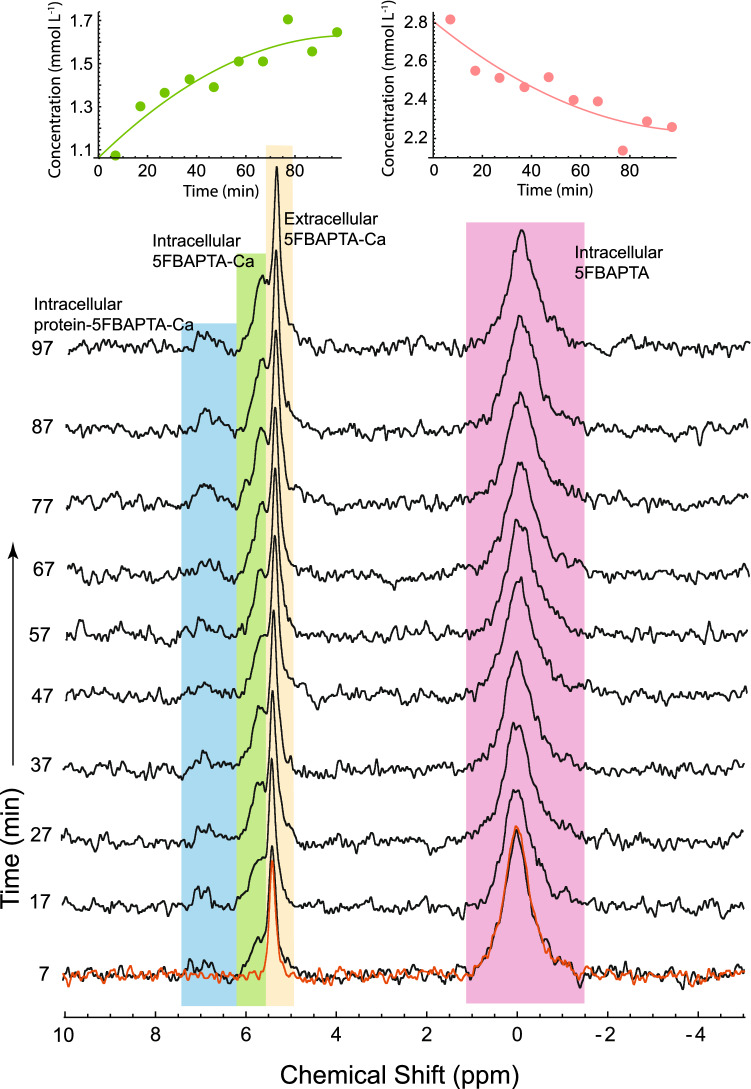
^19^F NMR spectra of RBCs loaded with 5FBAPTA and treated with Yoda1 in the presence of Ca^2+^, at 37 °C. The peak from free 5FBAPTA inside the RBCs (initially 4 mmol [L RBC]^−1^) is highlighted in pink; yellow highlights the peak from the extracellular 5FBAPTA-calcium complex; green, the peak from the intracellular 5FBAPTA-calcium complex; and blue, the peak from the intracellular protein-5FBAPTA-calcium complex. The sample was 0.5 mL RBCs (*Ht* = 0.73) in 154 mM NaCl and 10 mM d-glucose. The spectra were recorded every 10 min with NMR settings as for Fig. 1. The superimposed red spectrum at 7 min is from the 6th spectrum of a 1 h time course recorded with the RBCs in the presence of 2.0 µL 1 M CaCl_2_ (corresponding to 4.0 mM Ca^2+^ concentration averaged over the sample). Then, Yoda1 was added as 1.0 µL of 14 mM in DMSO; this value combined with the knowledge of the *Ht* value gave a concentration of 38 µmol [L RBC]^−1^. Left inset: combined integral of the peak from 5FBAPTA-Ca (green plus yellow). Right inset: integral of intracellular free 5FBAPTA (pink). The fitted equations and their parameter values were (left and right, respectively): 1.06 ± 0.07 + (0.011 ± 0.003) *t − *(0.00005 ± 0.0003) *t*^2^, and 2.81 ± 0.09 − (0.010 ± 0.004) *t − *(0.00005 ± 0.0004) *t*^2^.

Piezo1-mediated uptake of Ca^2+^ into RBCs was measured with the sample used for Fig. 2. The sample was supplemented with 4.0 mM CaCl_2_ and a spectrum was recorded (red spectrum superimposed by that at the 7 min mark in Fig. 3). Then, 1.0 µL of 14 mM Yoda1 in DMSO (making its concentration in the RBC suspension 38 µmol [L RBC]^−1^) was added, and ^19^F NMR spectra were recorded every 10 min for a total of ~ 100 min. The peak assigned to intracellular 5FBAPTA declined while the peaks that are highlighted in blue and green in Fig. 3 rose in an apparently exponential manner (fitted empirically by a quadratic in time), plateauing at ~ 90 min.

The prominent peak at ~ 5.5 ppm that changed little over the time course was present prior to adding the Yoda1 (the red spectrum in Fig. 3, as noted above). Therefore, this could be assigned to the extracellular complex of 5FBAPTA with Ca^2+^ (5FBAPTA-Ca) thus indicating some haemolysis. The peak retained approximately the same integral during the subsequent time course, while the peak to high frequency (intracellular 5FBAPTA-Ca) grew. Similarly, a poorly defined peak at ~ 7 ppm grew. This was subsequently assigned to a ternary complex of 5FBAPTA-Ca with protein (see Supplementary Information for the detailed argument).

In a separate experiment, the sample that had been supplemented with Ca^2+^ was centrifugally washed before a ^19^F NMR spectrum was recorded; this led to a significant reduction of the peak at ~ 5.5 ppm, thus confirming the assignment to extracellular 5FBAPTA-Ca (also see Supplementary Information Fig. S3 and Supplementary Discussion).

The initial rate of Ca^2+^ entry was estimated from fits of quadratic curves to the data (insets in Fig. 3 using the calibration of 4 mmol [L RBC]^−1^) to be 11 ± 3 µmol [L RBC]^−1^ min^−1^. In separate experiments, the rate of Ca^2+^ entry was recorded with increasing amounts of added Yoda1, while invoking more haemolysis, the net rate increased approximately linearly with the amount of Yoda1 added, up to twice the amount routinely employed.

### A23187 stimulated Ca^2+^ uptake (and compared with Yoda1)

**Figure 4:**
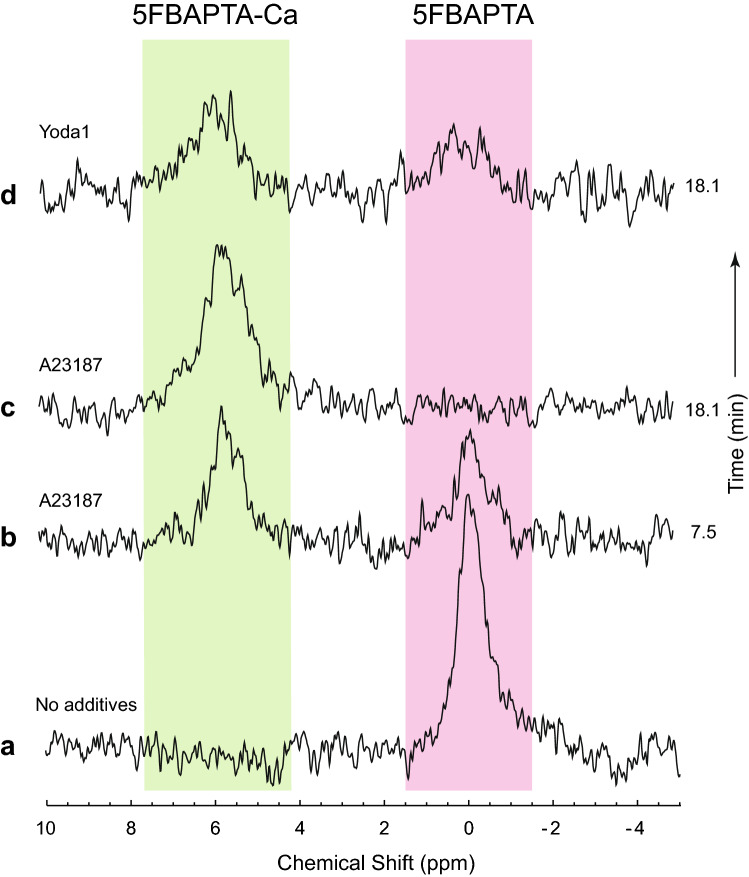
^19^F NMR spectra showing A23187 (and Yoda1 in comparison) stimulated uptake of Ca^2+^ in RBCs loaded with 5FBAPTA (4 mmol [L RBC]^−1^), at 37 °C. Pink highlights the resonance corresponding to intracellular free 5FBAPTA that was assigned the chemical shift δ = 0.0 ppm; and the green highlights the peak from the calcium complex that was centred at ~ 5.8 ppm. The sample of 0.5 mL RBCs (*Ht* = 0.62) was constituted in 154 mM NaCl, 10 mM glucose. (**a**) The RBCs had been loaded with 4 mM 5FBAPTA as described in Methods. Then, for (**b**,**c**), added with brisk mixing (by five-fold rapid inversion and re-inversion of the NMR tube) were: 5 µL 1 M CaCl_2_ making the concentration 10 mM averaged over the volume of the sample; and 0.5 µL 20 mM A23187 in DMSO giving a concentration of 32 µmol (L RBC)^−1^. For (**d**) 5 µL 1 M CaCl_2_ and 1.0 µL 14 mM Yoda1 in DMSO giving a concentration of 45 µmol (L RBC)^−1^ were mixed into the 0.5 mL susupension. The time indicated on the right of each spectrum was the mid-point of spectral accumulation after a 2 min lag between mixing the sample and starting FID accumulation. NMR settings were as for Fig. 1 except the time per spectrum was 10 min 47 s.

A conventional way of increasing the rate of Ca^2+^ entry into RBCs is with the ionophore A23187. It mediates uptake at a rate that relies on the Ca^2+^ concentration gradient across the plasma membrane^25,26^. We compared the relative rates brought about by Yoda1 and A23187. Figure 4a shows a spectrum from RBCs loaded with 5FBAPTA, which was similar to the corresponding spectra in Figs. 2 and 3, thus attesting to the reproducibility of the loading procedure.

Then, A23187 was added simultaneously with 10 mM CaCl_2_. Within a minute, the intracellular 5FBAPTA-Ca peak was already large (Fig. 4b), and the complexation reaction was complete by 18 min (Fig. 4c). Thus, all the intracellular 5FBAPTA had formed the Ca^2+^-complex. Hence, massive Ca^2+^ flux was achieved with this ionophore, as has been observed in other non-NMR experiments^26^. The poorly resolved emergent peak at ~ 5.5 ppm corresponded to extracellular 5FBAPTA-Ca but this was fortuitously not prominent here (as opposed to the much more obvious feature in Fig. 3 acquired from a different sample). With RBCs from the same loading procedure, treatment with an amount of Yoda1 the same as for the spectra in Figs. 3 and 4d revealed that after ~ 18 min over half of the 5FBAPTA had become complexed with Ca^2+^. In other words, the rate of entry of Ca^2+^ was slower than with A23187 for the particular concentrations chosen for the two reagents.

### ^13^C NMR of glycolysis

**Figure 5:**
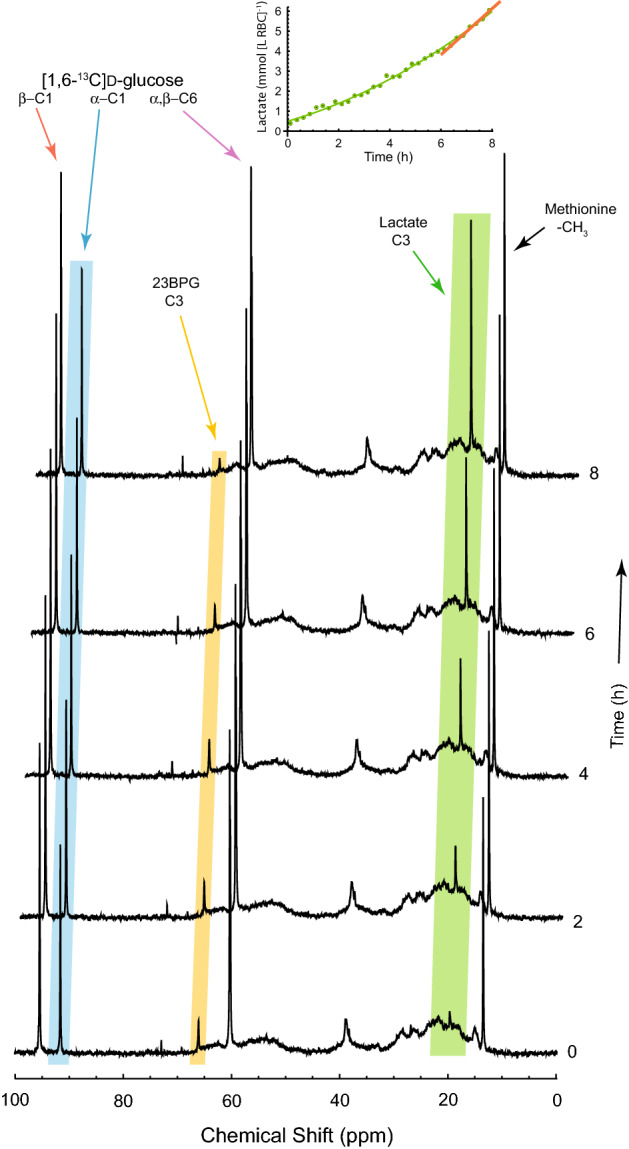
^13^C NMR (100.46 MHz) spectral time course of glycolysis in RBCs loaded with 5FBAPTA, at ~ 34 °C. 3 mL RBCs (*Ht* = 0.75) loaded with 5FBAPTA-AM were supplemented with [1,6-^13^C]d-glucose and ^13^CH_3_-l-methionine (final concentrations averaged over the sample volume were 8 mM and 7 mM, respectively). The times indicated on the right are relative to an arbitrary start time when the sample was removed from storage at 4 °C, after preparation 1 h before. Every eighth spectrum is shown. The inset shows the concentration of lactate corrected to account for *Ht*. A quadratic (0.489 + 0.368 *t* + 0.0398 *t*^2^; solid green) was regressed onto the full time course data set (green dots) that included the transient stage of the progress curve (see Discussion for why this routinely occurred), and the straight line ([−3.10 ± 0.84] + [1.15 ± 0.11] *t*; solid orange) was regressed onto the integrals from the more linear last 2 h (8 points). The labels indicate assignments to the main solutes: glucose; 2,3 bisphosphoglycerate (23BPG); lactate; and the chemical shift and intensity reference methionine (13.5 ppm). Green highlighting emphasizes the rising lactate resonance; orange, the emergence and then decline of 23BPG; and blue, the C1 atom of the α-anomer of glucose. (The other two glucose resonances are plain.) The integral of the methionine resonance remained constant, within experimental error. Spectra were recorded every 15 min. NMR settings: BBO probe; RF sampling pulse duration 13 µs based on a 90° pulse of 35 µs and estimated *T*_1_ of ~ 5 s hence the Ernst angle value; broadband gated decoupling of ^1^H was used to remove ^1^H–^13^C splitting in the spectra; the inter-FID delay was 2.84 s; 296 complex FIDs were summed per spectrum; a line broadening factor of 5 Hz was applied in exponential multiplication of the FIDs prior to Fourier transformation.

Having established a reproducible means of loading 5FBAPTA into RBCs and perfecting a means of supporting the cells metabolically in gelatin gels, it became important to establish the effect that 5FBAPTA might have on glycolysis both in RBC suspensions alone and in gels. 5FBAPTA-loaded RBCs were supplemented with [1,6-^13^C]d-glucose and time courses of ^13^C NMR spectra were recorded. An example from a suspension of RBCs (not in gel) is given in Fig. 5. The steady rise of the lactate resonance at 19.5 ppm is clear (green highlighting) and yet the inset graph shows an upward concavity of the progress curve.

The slope of the fitted curve in the last 2 h of the time course indicated a rate of 1.15 ± 0.11 mmol (L RBC)^−1^ h^−1^. This value was lower than the 2.06 mmol (L RBC)^−1^ h^−1^ recorded for a suspension of unloaded RBCs (not in gel; Supplementary Information Fig. S6) at ~ 34 °C, and 3 mmol (L RBC)^−1^ h^−1^ expected for 37 °C^1,27^.

## Discussion

### Basic experiment

Notwithstanding the low S/N in the ^19^F NMR spectra acquired in our first round of compression experiments (e.g., Fig. S1), there was consistently clear evidence of enhancement of the intracellular 5FBAPTA-Ca peak over time, relative to uncompressed controls. The S/N values were comparable, for a similar time of signal averaging, with earlier reports (e.g., fifth figure in^8^, eighth figure in^28^, and third and sixth figures in^10^). However, we sought refinement of methods to increase the S/N and hence the precision of quantification of flux rates from the low *Ht* values of ~ 0.2 that were dictated by using gels. Specifically, the gels are only stable against cracking with *Ht* values less than ~ 0.2.

The spectra shown in Fig. S1 were recorded after the RBCs has been stored at 20 °C for over 30 h. The original aim of recording ^19^F NMR spectral time courses of the 5FBAPTA-Ca peak could not be realised because of the low S/N. The next step towards increasing S/N was to record the spectra at 37 °C. This was only partially successful, but it included the loss of the ^19^F^-^ resonance (Fig. S3). This is explained by a reduction of hydrogen-bonding of the ^19^F^-^ ions to protein (presumably haemoglobin because of its overwhelming abundance) inside the cells. Hydrogen bonding has been described before as the chemical basis of the ‘split peak effect’ with ^19^F^-^ in RBC suspensions, whereas for the experiments relating to Fig. S3 it is the gelatin that interacts with the anion. Additionally, the signal from inside the RBCs was very low (and broad) because the intracellular volume was so relatively small due to the low haematocrit (*Ht* = 0.2).

### 5FBAPTA-AM loading

Quantification of the rate of Ca^2+^ uptake required knowledge of the concentration of 5FBAPTA inside the RBCs. This was provided by measuring the kinetics of conversion of 5FBAPTA-AM to the free anion. It was done by acquiring ^19^F NMR spectral time courses from 0.5 mL RBCs, to which 10 µL of 200 mM 5FBAPTA-AM in DMSO was added. Figure 2 shows a typical time course in which the only (albeit broad) resonance was from free 5FBAPTA. The measured rate (by regression analysis) was consistent with earlier reports that indicated loading times of 20 min to 1 h at 37 °C^8,10,11,28^.

When loading RBCs with Fluo-4, Cahalan et al.^18^ used a 1:1000 dilution of cells with 5 µM Fluo-4-AM. Assuming that all the dye partitioned into the RBCs (as it should have, based on our findings with 5FBAPTA), this implies a concentration of 5 mmol (L RBC)^−1^. This concentration is comparable to what we routinely used, thus making the extent of Ca^2+^ buffering similar in both studies.

### Yoda1 and A23187 stimulated Ca^2+^ influx

Yoda1 brought about influx of Ca^2+^ that occurred at a rate comparable to that achieved with a typically used concentration of A23187. Both rates were positively dependent on the effector concentration and occurred on a time scale much shorter than for the stretched/compressed RBC samples.

The sharp peak at ~ 5.5 ppm in the spectra in Fig. 3 was reduced if the cells were centrifugally washed in fresh saline. In fact, Murphy et al.^10^ observed a similar peak and successfully suppressed it with Eu^3+^ added to the suspension medium, thus confirming its extracellular location, since trivalent cations scarcely permeate the RBC membrane.

### ^19^F NMR peak assignments

Complexes of 5FBAPTA with different metal cations have a wide range of ^19^F NMR chemical shifts^8,29^. This range is due to the sensitivity of the ^19^F atom (attached to the two benzene rings in this case) to redistribution of electron density in the aromatic system. Because ^19^F atoms are also hydrogen bond acceptors (e.g.,^5,30,31^), it is expected that in the presence of high concentrations of amino acid side chains that the 5FBAPTA ^19^F NMR resonance would shift; the interaction is likely to be enhanced if two of the four carboxyl groups are neutralized by binding to Ca^2+^. This was born out by the appearance of the broad resonance at ~ 7 ppm in RBCs that had been loaded with 5FBAPTA and then made permeable to Ca^2+^ either with Yoda1 or A23187 (Figs. 3 and 4) but not in the absence of Ca^2+^ (Fig. 2). Furthermore, addition of K_4_5FBAPTA to gelatin gave a peak at the same chemical shift as extracellular 5FBAPTA-Ca attesting to the difference in this respect between gelatin (which is denatured collagen) and haemoglobin. The presence of some contaminating Ca^2+^ in the Gelita bovine gelatin is not entirely ruled out but previous atomic absorption spectrometry reported none (results not shown).

The detection of contaminating ^19^F^-^ in the gelatin was readily assigned by adding NaF, so the amount present could be quantified. It was not expected to have had any adverse effects on RBC metabolic or cation transport rates at the sub-millimolar concentrations that existed in the samples^32^. The disappearance of the ^19^F^−^ resonance (δ ~ 1.9 ppm; compare Fig. 1, and Figs. S3a and d) in gels, when warmed from 20 to 37 °C, is consistent with the weakening of hydrogen bonds to water and proteins that is known to occur with an increase in temperature. On the other hand if fast motional narrowing had been the dominant phenomenon then the signal might have persisted. The fact that ^19^F^−^ forms hydrogen bonds with water and proteins is also evidenced by the split peak effect seen with it in suspensions of RBCs^5,33^.

We base the interpretation of the temperature dependence of the peak we assigned to the protein-5FBAPTA-Ca complex on the same hydrogen bonding effect (see Figure S5). Specifically, there are two competing mechanisms that bring about ^19^F NMR spectral peak disappearance of the ^19^F-containing species: (1) hydrogen bonding of F-atoms to donor sites, and; (2) chemical exchange of the ^19^F-containing species between two or more binding sites. At lower temperatures hydrogen bonding is enhanced. Hence, at 20 °C the F-atoms of 5FBAPTA will bind more strongly to neighbouring molecules, with the most abundant being haemoglobin in the RBC. Since haemoglobin is relatively immobile the ^19^F NMR peak(s) will be broadened. The broadening is sufficiently great to make the peak indistinguishable from the noise in the spectra. On the other hand, elevation of temperature will enhance the rate of exchange between binding sites (where the chemical shifts are different) and in the case of ^19^F^−^ this gives rise to disappearance of the peak from tiny concentrations (like the amounts already present in commercial gelatin), whereas the exchange rate for the protein-5FBAPTA-Ca complex is not sufficiently great to cause exchange-broadening. This situation is coupled with fast-motional narrowing at the higher temperature thus making the peak clearly distinguishable from the baseline noise, at 37 °C.

### RBC glycolytic rate: 5FBAPTA loaded

The rate of glucose consumption in human RBCs at 37 °C is well characterized: at neutral pH with an adequate supply of glucose it is 1.6 mmol (L RBC)^−1^ h^−1^; and because of deflection of carbon atoms via the pentose phosphate pathway in which CO_2_ is released, this rate translates into ~ 3.0 mmol (L RBC)^−1^ h^−1^ of lactate production^34^. The rate measured in the control RBCs reported in Fig. S6 was ~ 30% less than this, which is attributed to the lower temperature used in these studies in which RF heating of the sample (due to the ^1^H decoupling used to remove ^1^H–^13^C splittings in the ^13^C NMR spectra) could not be ruled out. On the other hand, Beutler^27^ states that the ratio of the activity of hexokinase (the first and rate controlling step in human RBC glycolysis) at 30 °C/37 °C is 0.709 ± 0.037, which is consistent with our findings and suggests that the RF heating might have been less than predicted. Comparison with the lactate production rate by RBCs loaded with 5FBAPTA was what we sought. Since the conditions of the experiments (including the NMR methods) were the same, the rate comparison is valid. As seen from Fig. 5, the rate was 1.15 ± 0.11 mmol (L RBC)^–1^ h^−1^ compared with Fig. S6, 2.06 ± 0.12 mmol (L RBC)^−1^ h^−1^ at approximately the same stage (~ 7 h) in the time course; in other words, the rate was 56% of that of the normal control.

We propose that the lower rate is due to sequestration of Ca^2+^ and Mg^2+^ by the 4 mmol (L RBC)^−1^ 5FBAPTA inside the RBCs. Turnover of Ca^2+^ in RBCs occurs via Piezo1 and PMCA^24^. Hence, with extensive buffering of the concentration by 5FBAPTA in the loaded cells, the Ca-ATPase would be mediating less flux than normal. Mg^2+^ binding to 5FBAPTA is weaker than for Ca^2+^^8^ but its concentration in the cytoplasm is substantially higher so some sequestration by 5FBAPTA is expected. When this occurs, it will affect the concentration of Mg^2+^-ATP, the true substrate of the kinases including the three in glycolysis^27,35^. However the relative weakness of binding is underscored by the lack of effect of Mg^2+^ on the ^19^F NMR chemical shift of 5FBAPTA-Ca^8^.

Another modification to our previous protocol for preserving long term metabolic activity in RBCs was to use the insight provided by Kamp et al.^36^. They showed that the redox reagent DTE slows the development of echinocytes in such studies and they attributed this to inhibition of ATP-dependent phospholipid flippase activity. Therefore, the time courses reported in Figs. 5 and S6 were carried out in the presence of DTE.

In light microscopy examination of the samples (results not shown), there were no “spiky” echinocytes evident despite up to ~ 40 h of incubation at 20 °C. This was an important repeated finding because it is feasible that the greatly increased membrane curvature that occurs on formation of echinocytes could have affected the activity of Piezo1.

Overall, the RBCs loaded with 5FBAPTA continued to metabolise glucose, albeit at a reduced rate of 56% of the control. However, this was evidently sufficient to maintain RBC shape, and by implication the activity of the various ATP-dependent cation pumps.

The time courses of lactate production (insets in Figs. 5 and S6 are concave upwards for ~ 6 h, as has been reported and explained before^1^: the ^13^C atoms from the added [1,6-^13^C]d-glucose flush out the un-detectable ^12^C-containing metabolites (of the order of 5 mmol [L RBC]^−1^ 23BPG and ~ 1 mmol [L RBC]^−1^ of all others in the glycolytic and pentose phosphate pathways) that lead to ^12^C-l-lactate.

### Definitive experiments

The time courses of Ca^2+^ influx were necessarily conducted for more than 30 h, as was done previously when recording the effect on glycolytic rate of compressing gels containing RBCs^1^. Since the rate changes then were ascribed to enhanced Ca^2+^ entry, it was expected that the observations made with RBCs loaded with 5FBAPTA should also be of the same duration. The metabolic activity of the RBCs loaded with 5FBAPTA was maintained over these long times (Figs. 5 and S6).

In all experiments conducted with either stretching or compression, the control cells showed lower signals from the 5FBAPTA complexes. In calculating the influx rate, it was necessary to take into account not only the 5FBAPTA-Ca concentration but also that of the protein-5FBAPTA-Ca complex (Figs. 1, 3, S1 and S5). The existence of such a complex (or complexes) was discovered in our earlier work via so called *z*-spectra of 5FBAPTA in RBCs. In these studies, the resonances were not apparent in the ^19^F NMR spectra but their existence was revealed by transfer of magnetic saturation to the free 5FBAPTA peak when various frequencies of the spectrum were selectively irradiated^9^. This is now the basis of using chemical exchange saturation transfer (CEST) for measuring the concentration of various divalent cations that form complexes with 5FBAPTA, and its various analogues^29^.

The rates of Ca^2+^ influx were increased by ~ 100% in stretched (or compressed) samples and this is consistent with the previous metabolic enhancement; but the influx enhancement was much less than the corresponding ^133^Cs^+^ influx enhancement. This is consistent with knowledge that the rate of transport of Ca^2+^ via Piezo1 is much lower than for the alkali-metal monovalent cations^22^. However, it is important to note that the fluxes recorded in our studies were *net* fluxes. The Ca-ATPase would still have been ejecting Ca^2+^ and it would have been in competition with the sequestration of Ca^2+^ brought about by 5FBAPTA.

The net rates of ~ 9 to ~ 20 µmol (L RBC)^−1^ h^−1^ (Figs. 1 and S1) were recorded at 20 °C (the temperature used for the stretching and compression experiments). Concomitantly, glycolysis ran at ~ 1.2 mol (L RBC)^−1^ h^−1^ with stretching or compressing bringing about stimulation by a factor of ~ 2. This implies that the Ca^2+^ flux must be of this order of magnitude as well because glycolysis provides ~ 1.5 ATP molecules per molecule of lactate produced (e.g., ^35^) and Ca-ATPase hydrolyses one ATP molecule per Ca^2+^ ion pumped out of the cell. This tight stoichiometric relationship therefore implies a much higher total flux. Indeed, the rheometric studies by Larsen et al.^13^ with radio-tracing (that reports total fluxes) with ^45^Ca^2+^ found maximal rates of ~ 200 µmol (L RBC)^−1^ h^−1^ at 37 °C. Control (non-shear) passive fluxes were ~ 6 to 10 µmol (L RBC)^−1^ h^−1^ at 37 °C. Our net fluxes were estimated at 20 °C so taking into account a typical rate enhancement factor (called Q_10_ for enzyme systems) of 3, places them above the range of passive flux and in the mid-range of the values found in these previous rheological studies^13,14^.

The RBC distortion in the definitive experiments (Fig. 1) was induced by stretching the gel-RBC sample. Stretching turned out to be more reliable than the previously used compression. In the latter experiments, occasionally (unpredictably), the gel-RBC sample contracted away from the walls of the compressing silicone rubber tube. Therefore, we surmised that the effects of RBC distortion would have been lost once this occurred. On the other hand, by stretching the sample, the cross-sectional area of the sample is reduced while the sample is positively elongated independently of whether the sample adheres to the walls of the tube or not; and it can be held this way for up to 45 h.

It is worth noting though that the molecular asymmetry brought about in gelatin gels by compression is greater than that for stretching. In other words, if the 20 cm long silicone rubber tube is stretched by, say, 15 cm when the gel is still liquid, then allowed to set and then released, it causes compression. Alternatively, the gel can be allowed to set before the silicone tube and contents are stretched. The different treatments produce different and subtle effects in NMR spectra from guest molecules (e.g.,^37–40^). The details of these effects are not directly relevant here but the reduced distortion of RBCs in such gels is. The fact that the data from Fig. S1 showed a higher Ca^2+^ flux than for Fig. 1 is consistent with this molecular-environmental difference in gels compressed or stretched by the same amount.

### RBC shape preservation

One aspect of the experimental protocol used for preserving RBC shape over tens of hours used the finding of Kamp et al.^36^ that DTE reduces the rate of echinocyte formation. While we surmised that echinocytosis could alter membrane curvature and hence activate Piezo1, it is not yet established if this is the case. Such increased curvature even in an echinocyte is on a much larger length scale than that which is likely to affect channel gating. We think of these as local and global curvatures, respectively^41^. Given that Piezo1 is directly influenced by the surrounding membrane, it is much more likely that the change in leaflet composition can influence Piezo1 activity. For example, this channel can gate in lipids alone^42^, and polyunsaturated lipids can modify the channel kinetics^43^, as can cholesterol^44^. And, more importantly, there is even precedence for a flippase being directly involved in Piezo1 regulation in myoblasts^45^.

Therefore, we posit that it is more likely that changes in distribution of phospholipids via the specific flippase (and hence inhibition by DTE) and cholesterol via binding to BSA could regulate Piezo1 in the setting of our experiments rather than the global curvature of the echinocyte. This is an area that is ripe for study by using our gel-stretching-NMR approach.

### Conclusions

We report the development of an experimental protocol that enabled the quantification of the net rate of transport of Ca^2+^ ions into human RBCs when they were distorted by stretching or compression in gels. The enhanced fluxes were much less than those of ^133^Cs^+^ transport and lactate production, reported previously^1^, but they were nevertheless reproducible. We established optimal conditions for loading RBCs with 5FBAPTA with no haemolysis and confirmed previous reports on optimal times for this process. The emergence of a prominent ^19^F NMR peak that is consistent with a ternary complex of protein with 5FBAPTA-Ca was consistent with a previous report^9^, but it was striking that the peak was only very obvious after the RBCs had been haemolysed, and that the spectra were recorded at 37 °C but not at 20 °C. This suggests that inhomogeneous ^19^F NMR peak broadening was operating in the intact cells, and failure to resolve the peak and hence its area could lead to underestimation of net Ca^2+^ concentration if the spectra were recorded at 20 °C (Figure S5).

The measured net influx rates of Ca^2+^ that resulted from RBC deformation were small. On the other hand, fluxes mediated by the small molecule agonist of Piezo1, Yoda1, and the ionophore A23187 were large and readily quantified over minutes to several hours by using suspensions of RBCs.

More generally, the rates of Ca^2+^ transport are so low and the experiments so protracted that we propose that ^133^Cs^+^ should be used instead as a representative of all cations (mono- and divalent) in NMR studies of the relationship between RBC shape and Piezo1 activity. The transport rate can be enhanced fivefold by distorting the cells, and equilibrium is achieved in less than 1 h even at 20 °C and 100 mmol [L RBC]^−1 133^Cs^+^^1^. The exploration of the effects of altered phospholipid and cholesterol distribution in the membrane bilayer on Piezo1 activity in RBCs, as discussed above, is one area where ^133^Cs^+^ flux measurements could be valuable.

Overall, the present study ‘closed the loop’ on the hypothesis that it is Ca^2+^ that mediates the acceleration of glycolysis in RBCs in mechanically distorted gels by directly measuring the flux. A further area for exploration will be selective inhibition of Ca-ATPase (PMCA) to gain information on total (as opposed to net) flux of Ca^2+^ using the NMR procedures developed here.

### Research conduct

We confirm that all experiments were performed in accordance with relevant guidelines and regulations of The University of Sydney (Australia).

## Material and methods

### Experimental system

**Figure 6:**
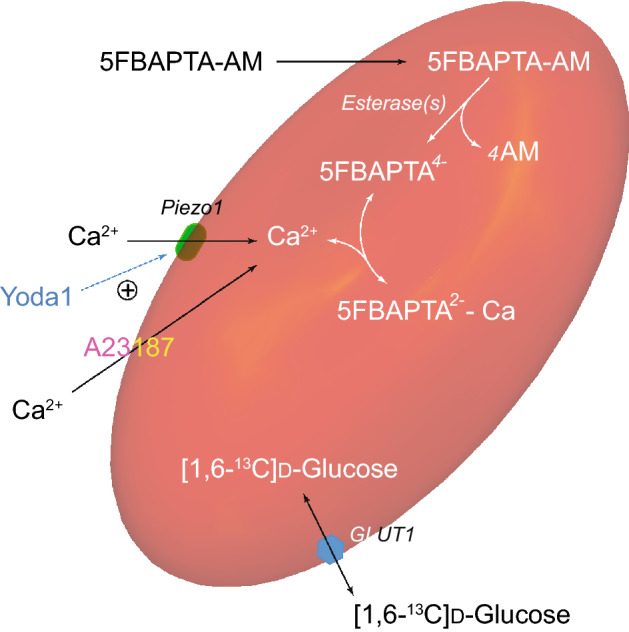
Graphical representation of an RBC under strain in a stretched gel (see below); it is loaded with the Ca^2+-^sensing chelator 5FBAPTA that yields separate ^19^F NMR signals from the free and Ca^2+^-complexed forms. Ca^2+^ enters via the mechanosensitive cation channel Piezo1 that can be activated (+ symbol) by the small-molecule compound Yoda1. Ca^2+^ entry into the RBC can also be mediated by the Ca^2+^-selective ionophore A23187. [1,6-^13^C]d-glucose enters the cell via the glucose transporter GLUT1; it was used in conjunction with ^13^C NMR spectroscopy to measure glycolytic flux under various experimental conditions. The model of the distorted RBC is based on Cartesian translation in *Mathematica*^46^ with the shape defined by the parametric equations given in^47^.

Figure 6 shows a schematic summary of the experimental system that was used to address the various questions posed in this study.

### Chemicals

Key reagents and their sources were: 5FBAPTA, Biotium (Landing Parkway, CA, USA); Yoda1, Glixx Laboratories (Hopkinton, MA, USA); and A23187, Sigma (Merck, Darmstadt, Germany). All other reagents and inorganic salts were AR grade.

### NMR

^13^C, ^19^F and ^133^Cs NMR spectra were recorded at 100.6, 376.42, and 52.48 MHz, respectively, on a Bruker (Karlsruhe, Germany) Avance III spectrometer with a 9.4 T vertical wide-bore magnet (Oxford Instruments, Oxford, UK). A 10-mm broadband observe (BBO) probe was used for all three nuclei; the tuning range of the outside ^1^H decoupler coil reached ^19^F, although the pre-acquisition delay (Bruker ‘de’) had to be altered from the typical 6.5 µs to 80 µs for optimal correction of a rolling baseline in the spectra. The probe temperature was calibrated (Bruker script ‘calctemp’ using a sample of neat methanol), and set to 20 °C for gel samples, and 37 °C for non-gel ^19^F samples/experiments; while for the ^13^C experiments, it was set to 30 °C to be conservative regarding RF heating due to broad-band ^1^H decoupling; the actual sample temperature was estimated to be ~ 34 °C. Spectra were processed using TopSpin 3.2 or 4.0 (Bruker), or read into MATLAB and *Mathematica* programs for baseline correction and nonlinear regression of mixed Gauss–Lorentzian line shapes to obtain relative peak integrals.

### RBCs

Blood (~ 35 mL) was aspirated using a syringe with attached 21 g needle from the cubital fossa of consenting volunteers, having signed an informed consent form for study participation. The study and form were approved by the University of Sydney Human Ethics Committee: Institutional Review Board (Project No. 2012/2882, Project Title: Magnetic resonance studies of red blood cell metabolism, biophysics, and cytology, and biochemical composition of plasma). Heparin (15 IU [mL blood]^−1^) was the anticoagulant, and the sample was centrifuged at 3000×*g* for 5 min at 20 °C. The supernatant and buffy coat were aspirated and discarded, and the resulting RBC pellet was resuspended in ~ 5 volumes of 154 mM NaCl containing 15 mM glucose. The sample was then re-centrifuged and the pellet of RBCs was suspended in ~ 5 volumes of saline and then bubbled for 5 min with CO to convert the heme-Fe(II) to a stable low-spin diamagnetic state (carboxyhaemoglobin) that gives optimal S/N in NMR spectra^5^. After re-centrifuging and aspiration of the supernatant, the RBC pellet was ready for the various experiments described herein.

Extents of haemolysis in various preparations were measured by comparing the colour intensity of a standard dilution of RBC lysate with the supernatant from the experimental sample using an Ernst Leitz (Wetzlar, Germany; No. 750) colorimeter with Olympus microscope light source.

### 5FBAPTA

*Theory of the method* 5FBAPTA provides unambiguous spectral assignments in the present context because of the absence of any endogenous fluorine-containing metabolites in human RBCs^10^. The Ca^2+^ complex of 5FBAPTA gives a ^19^F NMR resonance (peak) that is ~ 5.8 ppm at a higher frequency than the free form. The exchange between the two populations is slow on the timescale of the NMR experiment; in other words, the exchange rate constant is much less than the reciprocal of the frequency difference between the peaks of the free and Ca^2+^-complexed chelator. Thus, the ratio of the peak integrals, coupled with the reported thermodynamic dissociation equilibrium constant, *K*_d_, gives an estimate of the free Ca^2+^ concentration^8,10,48^:

1 $$\left[ {{\text{Ca}}^{2 + } } \right] = K_{{\text{d}}} \frac{{\left[ {5{\text{FBAPTA}} - {\text{Ca}}} \right]}}{{\left[ {5{\text{FBAPTA}}} \right]}}$$

where the ratio of the concentrations is that of the two peak integrals (for 5FBAPTA and 5FBAPTA-Ca), and *K*_d_ = 700 nM^10,48^ in RBCs. Thus, the free Ca^2+^ concentration is typically measured to be from ~ 60 to ~ 220 nM^48^.

The rate of exchange between the free and Ca^2+^-bound forms of 5FBAPTA is nevertheless sufficiently fast (295 s^−1^ at 37 °C) to allow application of chemical exchange saturation transfer (CEST) spectroscopy as an alternative NMR method for the estimation of free Ca^2+^ concentration^9^. As such, there is promise of selective quantification of other divalent cations based on the differential affinity of 5FBAPTA (and some of its analogues) for them^29^.

*Loading protocol* The 5FBAPTA-AM powder readily dissolved in DMSO to a concentration of 200 mM, which is ~ 4 times higher than the non-fluorinated parent compound. This was useful for minimizing the amount of DMSO that was necessarily added to RBC suspensions with the 5FBAPTA-AM. An aliquot of 3.5 mL RBC suspension (haematocrit; *Ht* ~ 0.85; measured as described below) was added to a 15 mL plastic centrifuge tube. Then, according to experimental requirements, either 12 mL of 300 mM sucrose, 15 mM glucose was layered onto the suspension, or the same volume of 154 mM NaCl, 15 mM glucose, (in some cases also 10 mM dithioerythritol; DTE), and 1% w/v bovine serum albumin was used. To clarify the supernatant, the sample was centrifuged at 3000×*g* for 5 min at 20 °C. The volume of added 200 mM 5FBAPTA-AM was 20–60 µL, depending on requirements, and it was dispensed into the supernatant followed by rapid five-fold inversion-and-reinversion of the tube, making a concentration of 2–4 mmol (L RBCs)^−1^. The reagent all partitioned into the RBCs. Specifically, a white ‘bloom’ of 5FBAPTA-AM instantly formed in the supernatant and this was rapidly mixed with the RBCs. The sample was incubated at 37 °C in a water bath. As shown in Fig. 2, the loading was complete in ~ 80 min at 37 °C, although 100 min incubation was routinely used. A ^19^F NMR spectrum of the supernatant showed no unreacted 5FBAPTA-AM.

### Gels

*Gels containing RBCs* 1.75 g of granulated bovine gelatin (Gelita, Brisbane, QLD, Australia) was suspended in 5 mL of 60 mM NaOH, 110 mM NaCl, and 10 mM KCl in a 50 mL disposable plastic centrifuge tube. Physiological pH (pH 7.2–7.4) was only achieved by this surprisingly high concentration of NaOH. The gelatin solution was heated to 80 °C for ~ 20 min, then it was centrifuged at ~ 2000×*g* for 20 s to remove air bubbles. An aliquot of freshly prepared RBCs was thermally equilibrated to 38 °C in a water bath. The *Ht* of the pellet was typically 0.85, measured with a disposable glass capillary in a Clements (North Ryde, NSW, Australia) centrifuge and measured with a Hawksley (Kent, UK) haematocrit reader. 2.0 mL of the RBC pellet was pipetted into liquid gelatin and mixed using a stainless-steel spatula for 1 min without introducing air bubbles. The gelatin-RBC mixture was drawn into a 25 cm long silicone rubber tube (Sims Portex, Hythe, Kent, UK; 7 mm o.d., 5 mm i.d.) via an attached 10 mL plastic syringe. The end of the tube was then sealed with a Delrin plug taking care to avoid introducing air bubbles. The loaded silicone rubber tube was inserted into the bore of a bottomless thick-walled glass NMR tube (New Era, Vineland, NJ, USA). For samples that were subsequently compressed, the silicone tube was stretched by 15 cm and held in that state by a custom-made Nylon thumbscrew, at the top of the glass tube. Once the gelatin had gelled, the stretched silicone tube was released gently to compress the contents (gel/RBCs). For stretched samples, these were cooled below 20 °C before stretching in the apparatus.

### Regression analysis and spectral graphics

Data-fitting to equations that are linear or nonlinear in the dependent variable(s), were carried out with *Mathematica*’s NonlinearModelFit^46^ that yields quality-of-fit statistics in addition to the fitted parameter values.

Spectra were processed in TopSpin 4.0 (Bruker) and imported into Adobe Illustrator to generate the graphics.

## Supplementary Information

Supplementary Information.

## Data Availability

All data needed to evaluate the conclusions in the paper are presented in this paper and/or the Supplementary Information. Additional data relating to the paper may be requested from the authors.

## Abbreviations

5FBAPTA: 1,2-Bis(2-amino-5-fluorophenoxy)ethane-*N,N,N′,N′*-tetraacetic acid
5FBAPTA-AM: Tetraacetoxymethyl ester of 1,2-bis(2-amino-5-fluorophenoxy)ethane-*N,N,N′,N′*-tetraacetic acid
ATP: Adenosine triphosphate
CEST: Chemical exchange saturation transfer
DMSO: Dimethylsulfoxide
DTE: Dithioerythritol
FID: Free induction decay
*Ht*: Haematocrit
NMR: Nuclear magnetic resonance
PMCA: Plasma membrane calcium ATPase
RBC: Red blood cell
S/N: Signal-to-noise ratio
Yoda1: 2-[5-[[(2,6-Dichlorophenyl)methyl]thio]-1,3,4-thiadiazol-2-yl]-pyrazine
