# Supplementary material for: Enhanced Ca2+ influx in mechanically distorted erythrocytes measured with 19F nuclear magnetic resonance spectroscopy
Source: Sci Rep. 2021 Feb 12;11:3749. doi: 10.1038/s41598-021-83044-z (PMC7881017; doi:10.1038/s41598-021-83044-z)
Supplement: Supplementary file 1 — Supplementary Information. [file 41598_2021_83044_MOESM1_ESM.pdf]

SUPPLEMENTARY INFORMATION for:

Enhanced Ca^{2+} influx in mechanically distorted erythrocytes measured with ^{19}F nuclear magnetic resonance spectroscopy

Philip W. Kuchel[1*], Konstantin Romanenko[1], Dmitry Shishmarev[2], Petrik Galvosas[3], and Charles D. Cox[4,5]

[1]*School of Life and Environmental Sciences, University of Sydney, Building G08, Sydney, NSW 2006, Australia*

[2]*John Curtin School of Medical Research, Australian National University, Canberra, ACT, Australia.*

[3]*MacDiarmid Institute for Advanced Materials and Nanotechnology, School of Chemical and Physical Sciences, Victoria University Wellington, Wellington, New Zealand*

[4]*Victor Chang Cardiac Research Institute, Darlinghurst, Sydney, NSW, Australia*

[5]*St Vincent's Clinical School, Faculty of Medicine, University of New South Wales, Sydney, NSW, Australia*

Supplementary Introduction

General

We provide here some context and motivation for studying Ca^{2+} and its signalling in metabolism and cell shape by using NMR spectroscopy in the way that we developed in the current project.

Calcium

Mixed phosphates and hydroxides make up ~99% of the total calcium content of the human body. It is remarkable that in the face of this huge pool, it is free Ca^{2+} *inside* cells (ionically dissociated and in the micromolar-nanomolar concentration range) that regulates metabolism, and the organization of cytoskeletal networks.

In human blood plasma, the ~2.2 mM Ca^{2+} is distributed as 45% free, 45% bound to protein, and 10% bound to various ligands. Again, it is the free cation in *plasma* that has various signalling roles in cells that are perfused by it[1]; e.g., activation of phagocytosis in neutrophils is driven by Ca^{2+} uptake into their cytoplasm[2]. Based on extensive work, Lew and Tiffert have identified amongst other effects on RBC ionic homeostasis[3], the key role of Ca^{2+} influx in human RBCs during their senescence (and by analogy, in other mammals)[4]. Consequently, the methods developed in the present work will aid the understanding of factors that affect the role of Ca^{2+} in RBC shape, flexibility, and survival in the circulation.

Supplementary Results Part 1

Direct experiment

Figure S1 shows ^{19}F NMR spectra obtained from a sample of RBCs that had been loaded with 5FBAPTA and then compressed in gelatin gel, as used previously when enhanced glycolysis was discovered[5]. Figure S1c was from a control sample that had been prepared identically but not compressed, while Fig. S1b shows a superposition of the two spectra. The spectra clearly show a level of S/N like that reported by others; they imply the need to refine the method of measuring Ca^{2+} influx to improve the precision of the rate estimates.

The spectrum in Fig. S1a was recorded in 1.5 h at 20°C after the sample (RBCs and gelatin) had been compressed and stored for 17 h (overnight) at 4°C and then maintained at 20°C for 32.75 h. There are three main spectral features on which to focus (apart from the noise): (1) the broad peak with S/N = 3.6 and width-at-half-height ($\Delta\nu_{½}$) = ~500 Hz was assigned to free 5FBAPTA inside the RBCs and designated a chemical shift (δ) = 0.0 ppm; (2) the peak

located at δ ~ 1.76 ppm to lower frequency with S/N = 4.74 and $\Delta\nu_{½}$ = ~80 Hz was assigned to F^- despite the fact that none of this anion was separately added to the sample; and (3) the broad peak at ~6 ppm with S/N = 2.3 and $\Delta\nu_{½}$ = ~500 Hz was assigned to the intracellular Ca^{2+} complex of 5FBAPTA (5FBAPTA-Ca).

The ratio of the peak integrals of 5FBAPTA-Ca to free 5FBAPTA was ~0.19:1.0. Given that the loaded amount of 5FBAPTA-AM was 4.0 mmol (L RBC)$^{-1}$, the concentration of the complex was deduced from the integral ratio to be 0.64 mmol (L RBC)$^{-1}$. Since this complex primarily accumulated over 32.75 h (ignoring the overnight storage at 4°C), the *net* influx rate of the Ca^{2+} was calculated to be ~20 μmol (L RBC)$^{-1}$ h^{-1}. (In the Discussion, we present caveats associated with this estimate, and its relationship to the operation of the RBCs at 37°C.).

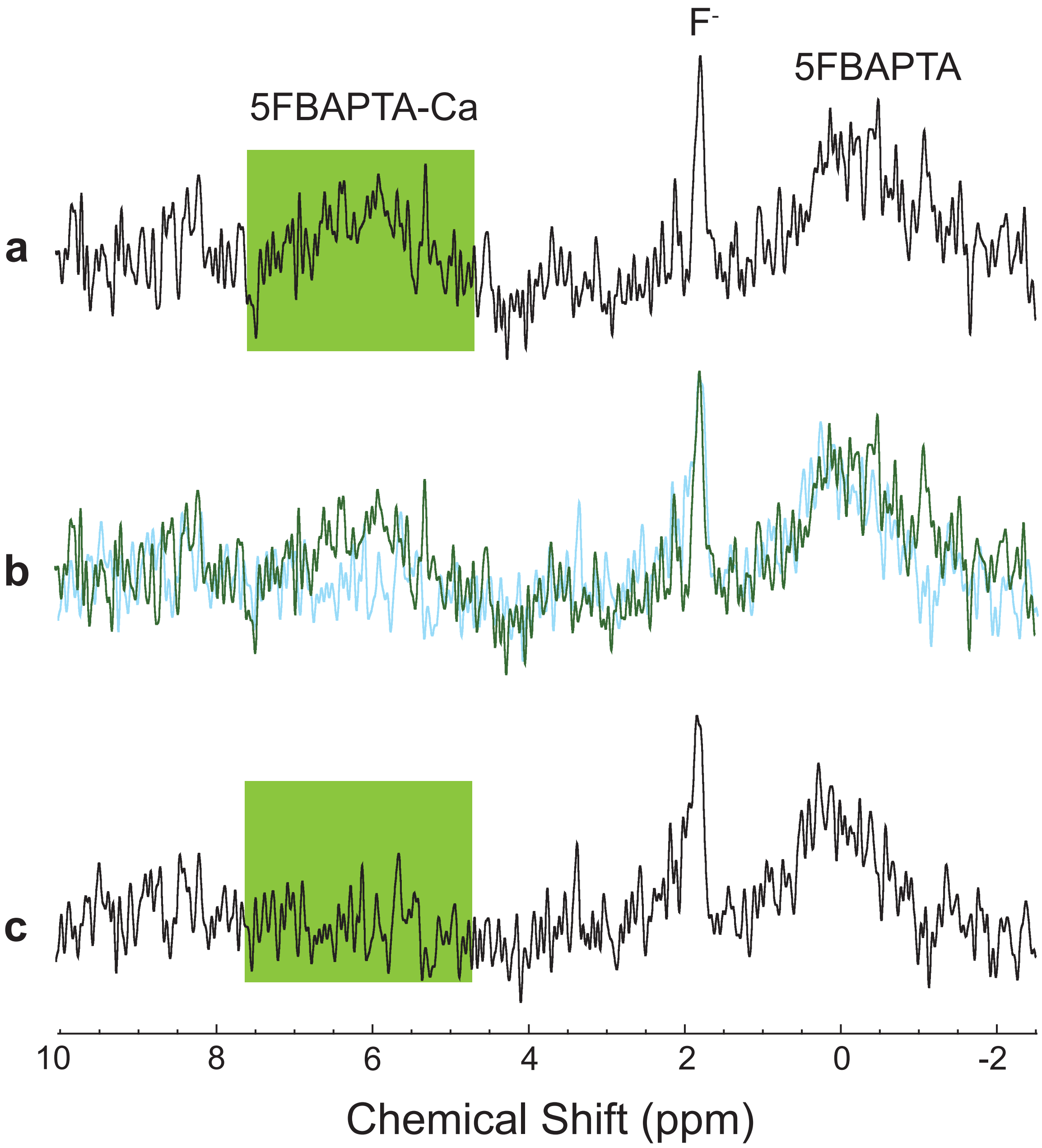

Figure S1. ^{19}F NMR (376.46 MHz) spectra of RBCs that had been loaded with 5FBAPTA and compressed by 75% in gelatin gel (35% w/v) at 4ºC for 17 h, and then maintained at 20ºC. For the sample that gave spectrum **a,**

the duration of compression at 20°C was 32.75 h; and for the relaxed control sample **c** it was 34.25 h. The green highlighting around ~6 ppm identifies the Ca^{2+} complex of 5FBAPTA inside the RBCs; the broad peak arbitrarily set to a chemical shift of 0.0 ppm is from free 5FBAPTA; and the sharper peak at ~1.9 ppm was assigned to free F^- from the gelatin. **b** shows the superposition of **a** (green) and **c** (blue). Sample details: 3.5 mL of RBCs (*Ht* = 0.80) incubated in 300 mM sucrose and 15 mM glucose, with 60 μL of 200 mM 5FBAPTA-AM in DMSO giving a total concentration of 4 mmol (L RBC)$^{-1}$. Two mL of these cells (*Ht* = 0.82) were added to 1.75 g of bovine gelatin (Gelita) in 5 mL of the NaOH/saline solution as described in Materials and Methods, giving a packing density (taking into account the partial specific volume of gelatin = 0.71[6]) of 0.20. NMR settings: BBO probe; 90° RF pulse duration was 35 μs; 1024 FIDs per spectrum; inter-transient delay 5 s contributing to a total time of spectral accumulation of 1.5 h; spectral width was 10 kHz (26.56 ppm); and FIDs were modified with 20 Hz line broadening by exponential multiplication.

Supplementary Methods

Because of the very low S/N (~4:1) in the ^{19}F NMR spectra of RBCs loaded with 5FBAPTA, when they were suspended in gelatin, a protocol was devised that raised this to ~15:1. This meant that we had to forego the aim to record spectral time courses of the influx of Ca^{2+} from a single sample held in the NMR spectrometer in the 10 mm probe, over 10's of hours. Signal enhancement was ultimately achieved by removing the RBCs from the gelatin, washing them centrifugally, and recording spectra in a 5-mm NMR probe that had substantially higher sensitivity. Specifically, for a 1 M solution of $^{19}F^-$ in the 10 mm probe (glass 10-mm NMR sample tube) and the optimized (dedicated for ^{19}F) 5 mm probe the S/N was approximately the same. The 10 mm sample volume was typically 6-10 times greater than for the 5 mm probe for which the volume was much smaller, so if the same *amount* of solute were present in each sample then the optimized ^{19}F NMR probe would give ~6-10 times the S/N. In addition, the RBCs in the gels had maximum packing densities of ~20% (*Ht* = 0.2), so extraction of them from the gel enabled the *Ht* to be raised four-fold to ~0.8. Moreover, the washing procedure removed any extracellular 5FBAPTA-Ca complex that could have formed if there had been haemolysis, thus avoiding mis-assignment of peak area to intracellular 5FBAPTA-Ca. Recording the spectra at 37°C, which was not feasible if retention of gel was required, meant that the extraneous $^{19}F^-$ resonance from the gelatin (Fig. S3) was also removed. Overall, the protocol used for extracting the RBCs from compressed or stretched gels after prolonged time courses used to assess Ca^{2+} influx is shown in Fig. S2, with the various steps described in the caption.

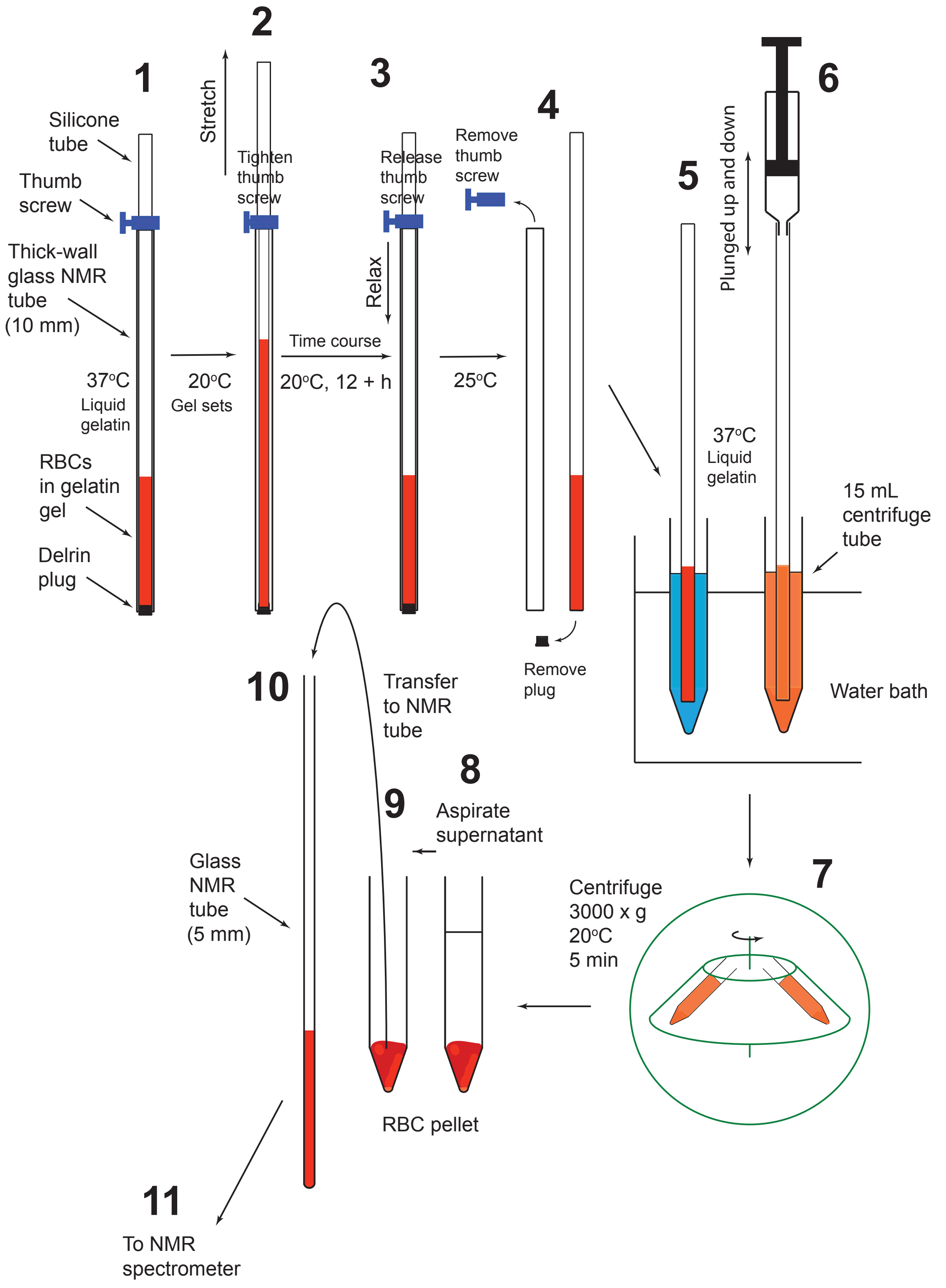

Figure S2. Experimental protocol for studying stretched (or compressed) RBCs that were extracted from the gel *prior to* ^{19}F NMR spectroscopy. The RBCs were loaded with 5FBAPTA, suspended in gelatin that was drawn into a silicone rubber tube that was held inside a glass NMR tube (**Step 1**). The silicone tube and its contents

were held compressed or stretched for up to 45 h at 20°C (**Steps 2** and **3**). At the end of the time course, the sample was removed from the stretching/compression device (**Step 4**) and then the contents of the silicone tune were extracted by melting the gel at 37°C and flushing with ~10 volumes of warm saline (**Steps 5** and **6**). The RBCs were separated from the liquid gelatin by centrifugation (**Steps 7 - 9**) including resuspension and re-centrifugation in warm saline. The RBC pellet was then transferred to a 5-mm NMR tube (**Step 10**) for acquisition of a ^{19}F NMR spectrum (**Step 11**).

Supplementary Results Part 2 and Discussion

Resonance assignment to $^{19}F^{-}$

The relatively sharp peak at 1.53 ppm (relative to 5FBAPTA) in the ^{19}F NMR spectra shown in Fig. S3 was unexpected and demanded assignment. Assignment was made by recording a ^{19}F NMR spectrum from a sample of gelatin alone. This was done first at 20°C, the same temperature as used for the series of stretched and compressed RBC experiments.

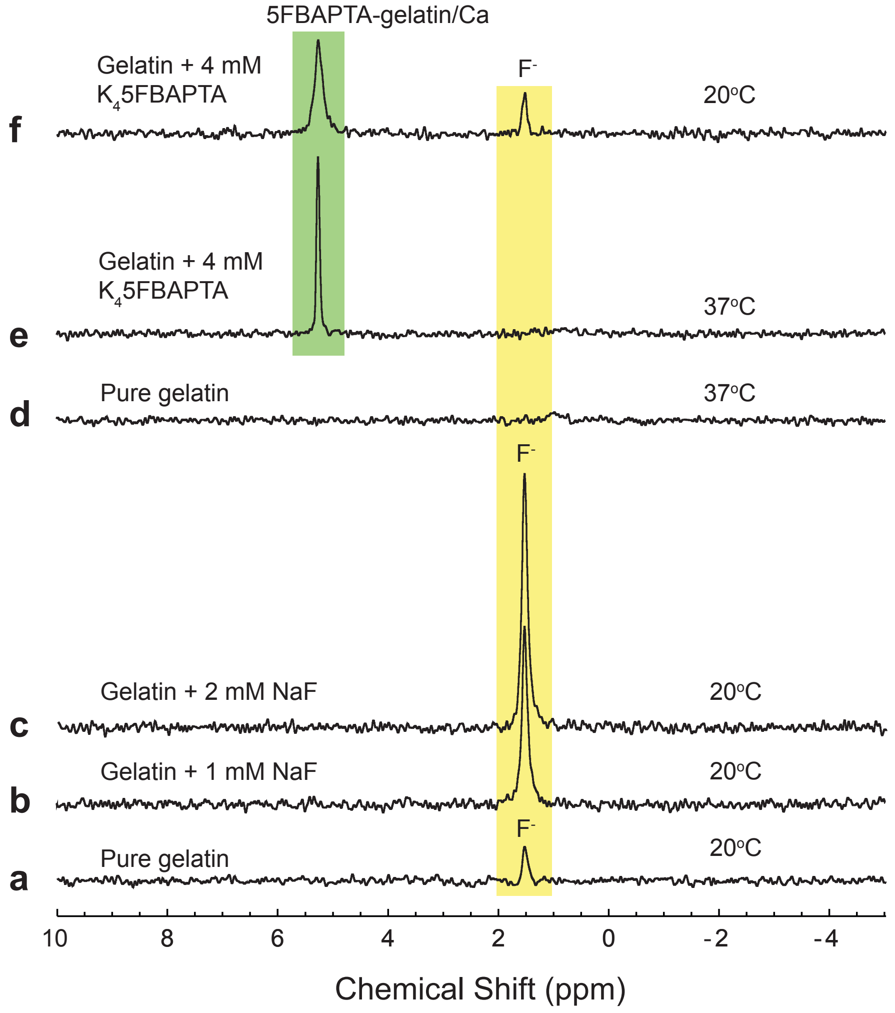

Figure S3. Assignment of the resonance at 1.53 ppm (relative to free 5FBAPTA) in ^{19}F NMR spectra of samples made up in 35% w/v Gelita gelatin, at 25 °C and 37°C. Yellow highlighting on F^{-}; green, on the

5FBAPTA-Ca complex. **a**, 0.7 mL sample of gelatin gel at 20°C; **d**, at 37°C; **b**, addition of 1 mM NaF to **a**; **c**, after addition of extra 1 mM NaF (making 2 mM); **e**, 0.5 mL sample with addition of 4 mM $K_4$5FBAPTA (10 µL of 200 mM) and ; **f**, sample **e** cooled to 20°C. NMR settings: 5-mm ^{19}F probe; total time per spectrum, 10 min; other parameters as for Fig. 1.

It is clear from Fig. S3a that the peak at 1.53 ppm was present in the spectrum from pure gelatin; and that addition of NaF to the sample after it was melted at 37°C and then reset, enhanced the peak. Further addition of NaF led to a direct quantitative increase in peak intensity, thus confirming the assignment to $^{19}F^-$. In other words, the Gelita gelatin that we used in all the experiments herein, and previously[5], is contaminated with F^-.

From the relative integrals of the spectra in Figs. S3a-c it was calculated that the concentration of $^{19}F^-$ in 35% w/v Gelita gelatin was 0.16 mmol L^{-1}. This implied a ratio of amounts of $^{19}F^-$:gelatin of 27 ppm.

While food standards for levels of F^- in gelatin vary from country to country, web searching revealed that this is within acceptable limits of all recommendations reported there. In other words, our finding of a $^{19}F^-$ resonance in the ^{19}F NMR spectra of 5FBAPTA loaded RBCs in gelatin gels could have been expected; it was due to the gelatin and not to a contaminant in the 5FBAPTA-AM or in the RBCs and the various preparation media.

Resonance assignment to extracellular 5FBAPTA-gelatin

Figure S3e shows a spectrum from gelatin that was supplemented with 4 mM $K_4$5FBAPTA and recorded at 37°C. Note that there was no added Ca^{2+} but the chemical shift was that of 5FBAPTA-Ca. Addition of extra Ca^{2+} (spectrum not shown) did not alter the chemical shift. When the sample from Fig. S3e was cooled to 20°C, the $^{19}F^-$ resonance appeared (as for Fig. S3a) and the peak from the $K_4$5FBAPTA was broadened.

The latter was concluded to be due to enhanced bonding of the F-atoms on the 5FBAPTA to hydrogen bond donors on the gelatin across a distribution of sites rather than due to increased exchange with free 5FBAPTA because the temperature had been lowered and not raised.

In summary: (1) gelatin contains F^- that gives a peak only when the temperature is lowered to ~20°C, consistent with hydrogen bonding to gelatin's amino acid side chains; while at 37°C these bonds are weakened, and exchange broadening occurred leading to loss of the peak into the baseline noise. And (2), 5FBAPTA forms hydrogen bonds with gelatin that means that its chemical shift is the same as for the complex with Ca^{2+}. Hence, if 5FBAPTA loaded RBCs in a gelatin gel rupture, a ^{19}F NMR peak that results from the release on 5FBAPTA to the

extracellular medium will be due to a combination of 5FBAPTA-gelatin and 5FBAPTA-Ca and possibly also a ternary complex of both species.

Resonance assignment to extracellular 5FBAPTA-Ca

Figure S4a shows a ^{19}F NMR spectrum of RBCs that had been loaded with 5FBAPTA, washed with saline (154 mM NaCl) and 1% w/v BSA, and supplemented with Yoda1. There was no Ca^{2+} in this medium. The small peak at 5.9 ppm was identified as intracellular 5FBAPTA-Ca, while the adjacent sharper one at 5.5 ppm according to the argument above (Fig. S3) was from 5FBAPTA-BSA and any 5FBAPTA-Ca that would have been released by haemolysis that occurred during and after the formation of the RBC pellet.

Figure S4b was recorded after Ca^{2+} was added to the washed RBCs and it gave rise to the sharp peak at 5.5 ppm that was consistent with formation of 5FBAPTA-Ca outside the RBCs, due to haemolysis. The emergence of the broad peak at 5.9 ppm was consistent with Ca^{2+} entry via Piezo1 and the formation of intracellular 5FBAPTA-Ca with a concomitant decline in the intracellular 5FBAPTA peak at 0.0 ppm. In Figs. S4c and S4d not only did the intracellular 5FBAPTA-Ca peak grow over time, but another peak at 7.3 ppm did as well; while the sharp peak at 5.5 ppm (assigned to extracellular 5FBAPTA-Ca) did not change in intensity. The latter implied that haemolysis had occurred during sample transfer to the 5-mm NMR tube and on subsequent mixing of the RBCs with the Yoda1 stock solution. The assignment of the peak at 7.3 ppm was concluded to be a ternary complex of Ca^{2+}, 5FBAPTA and intracellular protein as its growth occurred in parallel with that of 5FBAPTA-Ca (as is also evident in Fig. 3), and the concomitant decline of free 5FBAPTA inside the cells. Furthermore, addition of $K_4$5FBAPTA to the RBC suspension gave rise to a marked increase in intensity of the peak at 5.5 ppm, underscoring the veracity of the assignment of this peak to extracellular 5FBAPTA-Ca, concluded from Fig. S3. In conclusion: (1) in a simple suspension of RBCs, the sharp peak at 5.5 ppm was from extracellular 5FBAPTA-Ca; (2) in the presence of a high concentration of gelatin, this peak could also be due to 5FBAPTA-gelatin complex(es); (3) the peak at 5.9 ppm was from intracellular 5FBAPTA-Ca; and (4) the peak at 7.3 ppm was assigned to a ternary complex of protein-5FBAPTA-Ca, where, based on its high abundance, this was likely to have been haemoglobin. This is now discussed in more detail.

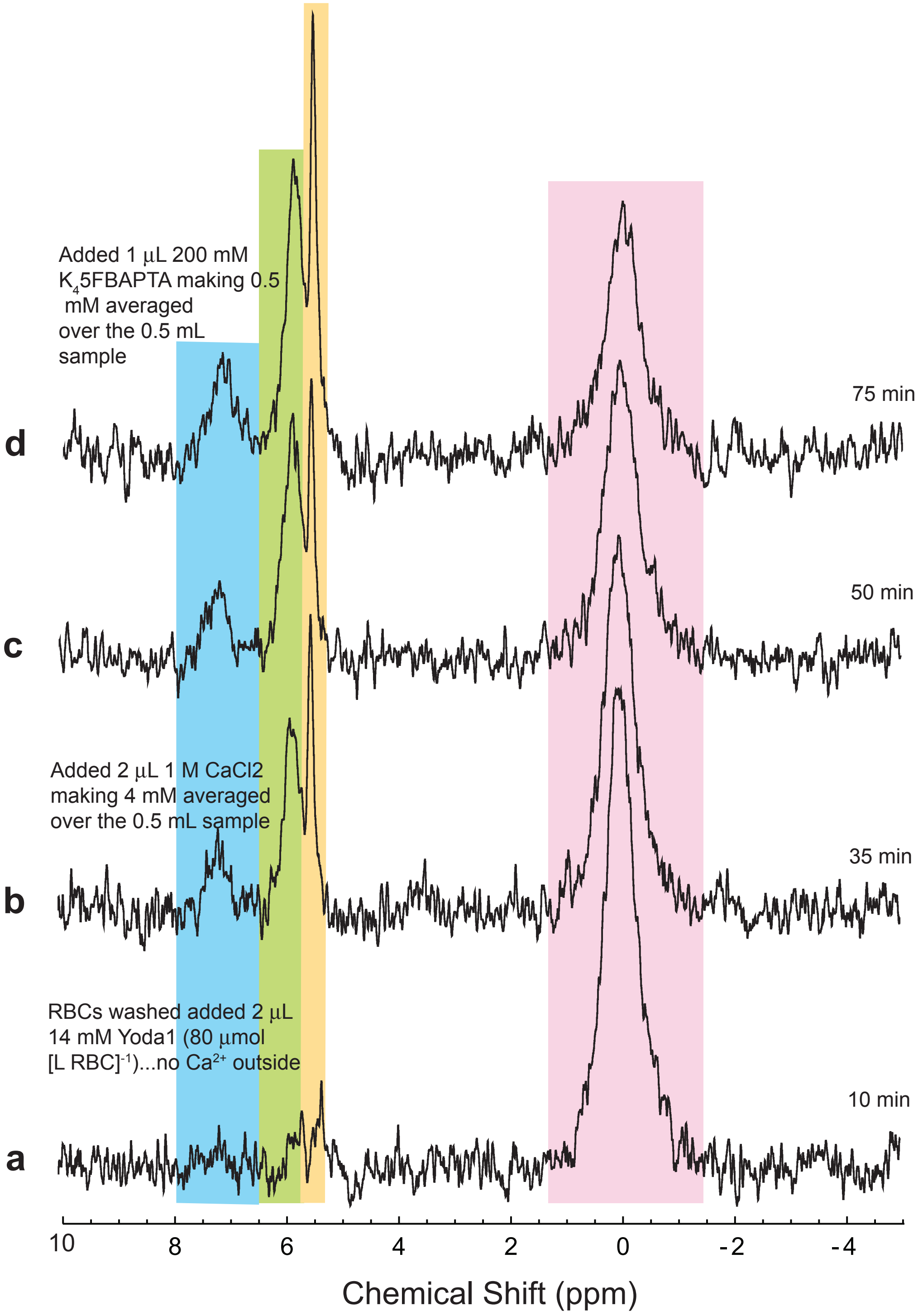

Figure S4. Assignment of ^{19}F NMR spectral peaks from 5FBAPTA and its complexes in RBC suspensions. The colour code is the same as used in Figs. 1-3. RBCs were loaded with 3.5 mM 5FBAPTA (see Methods) and washed in physiological saline (final *Ht* ~0.7) that was then supplemented with 80 µmol (L RBC)$^{-1}$ Yoda1,

giving spectrum **a**. For **b**, Ca^{2+} was added to sample **a** to give 4 mM averaged over the whole sample being well in excess of the 5FBAPTA. **c** was recorded from the sample 25 min later; and then $K_4$5FBAPTA was added (0.5 mM averaged over the sample volume) giving spectrum **d**. NMR settings: 5-mm ^{19}F probe; total time per spectrum, 10 min; other parameters as for Figs. 1, S1 and 3.

Resonance assignment to intracellular protein-5FBAPTA-Ca

Figure S5 shows the spectra acquired in an experiment designed to underscore the assignment of the peak at ~7.3 ppm. First, RBCs were loaded with 4 mmol $[L\ RBC]^{-1}$ of 5FBAPTA and the spectrum (Fig. S5a) was recorded with the sample at 20ºC. Only the single broad resonance (width at half the peak height; $\Delta\nu_{1/2}$ = 230 Hz) assigned to free 5FBAPTA was evident, as can also be seen in the spectra in Figure 2 that were recorded at 37ºC with slightly smaller $\Delta\nu_{1/2}$ values of ~200 Hz. This is consistent with the higher temperature causing motional narrowing of the peak. Second, the RBCs were lysed by freezing and thawing in liquid nitrogen and spectrum b was recorded also at 20ºC. No new peaks emerged and the only change was a slight narrowing of the peak at 0 ppm (free 5FBAPTA) to $\Delta\nu_{1/2}$ = 180 Hz. This change can be ascribed to the sample being magnetically more homogenenous because of the freezing and thawing cycle. In other words, there would no longer have been gradients of magnetic susceptibility across the cell membranes. Third, the haemolysate was supplemented with Ca^{2+} (~10 mM) to form 5FBAPTA-Ca. Figure S5c shows the resulting spectrum that had no evidence of free 5FBAPTA but the presence of the relatively sharp peak at ~5.7 ppm (as also evident in Figures 1, 3, 4 and S4) was consistent with assignment to the 5FBAPTA-Ca complex as seen in Figure S3e and f. Furthermore, a smaller peak at ~7.3 ppm, with an integral ratio of 0.2:1.0 relative to the adjacent peak from 5FBAPTA-Ca, appeared as well. Because of its appearance only after adding Ca^{2+} it was concluded to be a complex of Ca^{2+} with 5FBAPTA that was in a different molecular environment. It is important to note that this spectrum was recorded with the sample at 37ºC. Fourth, when the sample was cooled to 20ºC the spectrum lost the peak at ~7.3 ppm. Fifth, the latter peak, however, reappeared when the same sample was warmed back to 37ºC with almost no change in spectral resolution (line width) and relative intensities. The appearance and disappearance of the peak at ~7.3 ppm with cycling the temperature of the sample was reproducible in many similar experiments.

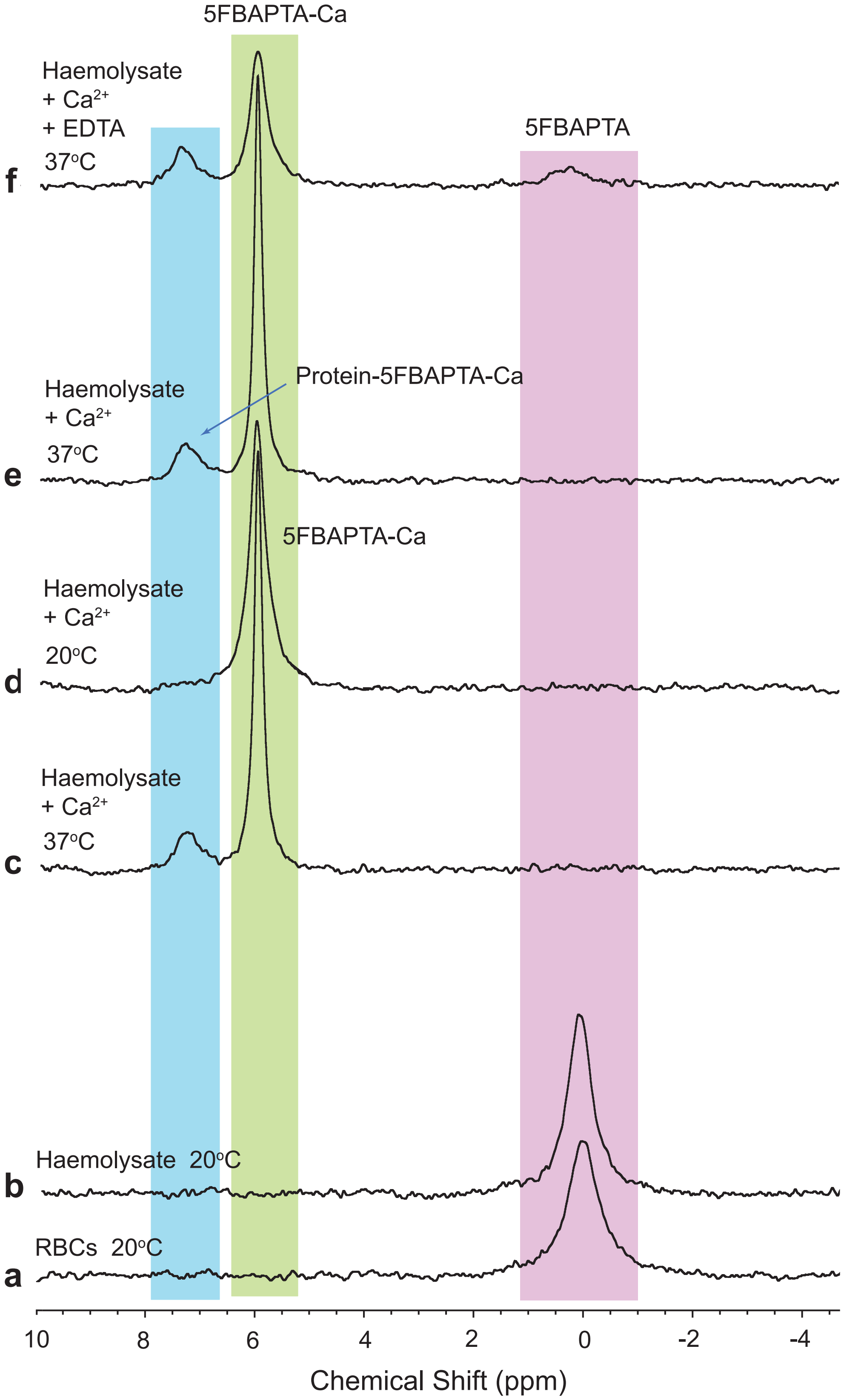
5FBAPTA-Ca
Haemolysate
+ Ca2+
+ EDTA
37oC
f
5FBAPTA
Protein-5FBAPTA-Ca
Haemolysate
+ Ca2+
37oC
e
5FBAPTA-Ca
Haemolysate
+ Ca2+
20oC
d
Haemolysate
+ Ca2+
37oC
c
Haemolysate 20oC
b
RBCs 20oC
a
10
8
6
4
2
0
-2
-4
Chemical Shift (ppm)

Figure S5. Assignment of the ^{19}F NMR peak at ~7.3 ppm to a protein-5FBAPTA-Ca complex; and the reversible dependence of its presence or absence on temperature. RBCs were loaded with 5FBAPTA as described in Methods. **a**, intact RBCs, spectrum recorded at 20ºC; **b**, the same sample after haemolysis by freezing and thawing in liquid nitrogen, spectrum recorded at 20ºC; **c**, same lysed RBC sample with added Ca^{2+}, and the spectrum was recorded at 37ºC; **d**, same sample as for **c** but recorded at 20ºC;**e**, same sample as for **c** and **d** but recorded at 37ºC; **f**, same sample as for **c**, **d**, and **e** also recorded at 37ºC but with added 10 mM EDTA. NMR: 5-mm ^{19}F probe; with 0.4 mL samples; acquisition parameters were as for Fig. 1 with 128 FIDs summed per spectrum; a line broadening factor of 20 Hz was applied prior to Fourier transformation.

As described in detail in the Discussion, this effect is attributed to hydrogen bonding of 5FBAPTA to the abundant much less mobile macromolecules in the RBC, especially haemoglobin giving rise to a an increased rotational correlation time and hence a reduced transverse relaxation time, T_2. The conclusion that it involves a co-complex with Ca^{2+} was based first on the appearance of the peak at ~7.3 ppm only after adding Ca^{2+}; and also by the experiment in Figure S5f. For this spectrum the sample was further supplemented with the divalent-cation chelator EDTA (ethylenediaminetetraacetic acid). It displaced the 5FBAPTA binding reaction to give rise to free 5FBAPTA, as seen by the re-appearance of the peak at 0 ppm while the peaks at ~7.3 and 5.9 ppm showed decreased intensity. This suggested that the EDTA was abstracting Ca^{2+} away from 5FBAPTA-Ca and also whatever was giving rise to the peak at ~7.3 ppm. Therefore, we concluded that the peak was due to 5FBAPTA-Ca that was in a molecular environment that was different from free solution (see Figure S3) or in cytosolic water space. We further concluded that the most likely new environment was that of a protein complex with 5FBAPTA-Ca; and in the RBC since haemoglobin constitutes ~97% of the protein in the cytoplasm it would be the most likely candidate.

^{13}C NMR of glycolysis

Figure S6 shows a ^{13}C NMR time course of spectra obtained from RBCs that had been loaded with 5FBAPTA and to which [1,6-^{13}C]D-glucose was added. The insert shows the corresponding progress curve for L-lactate.

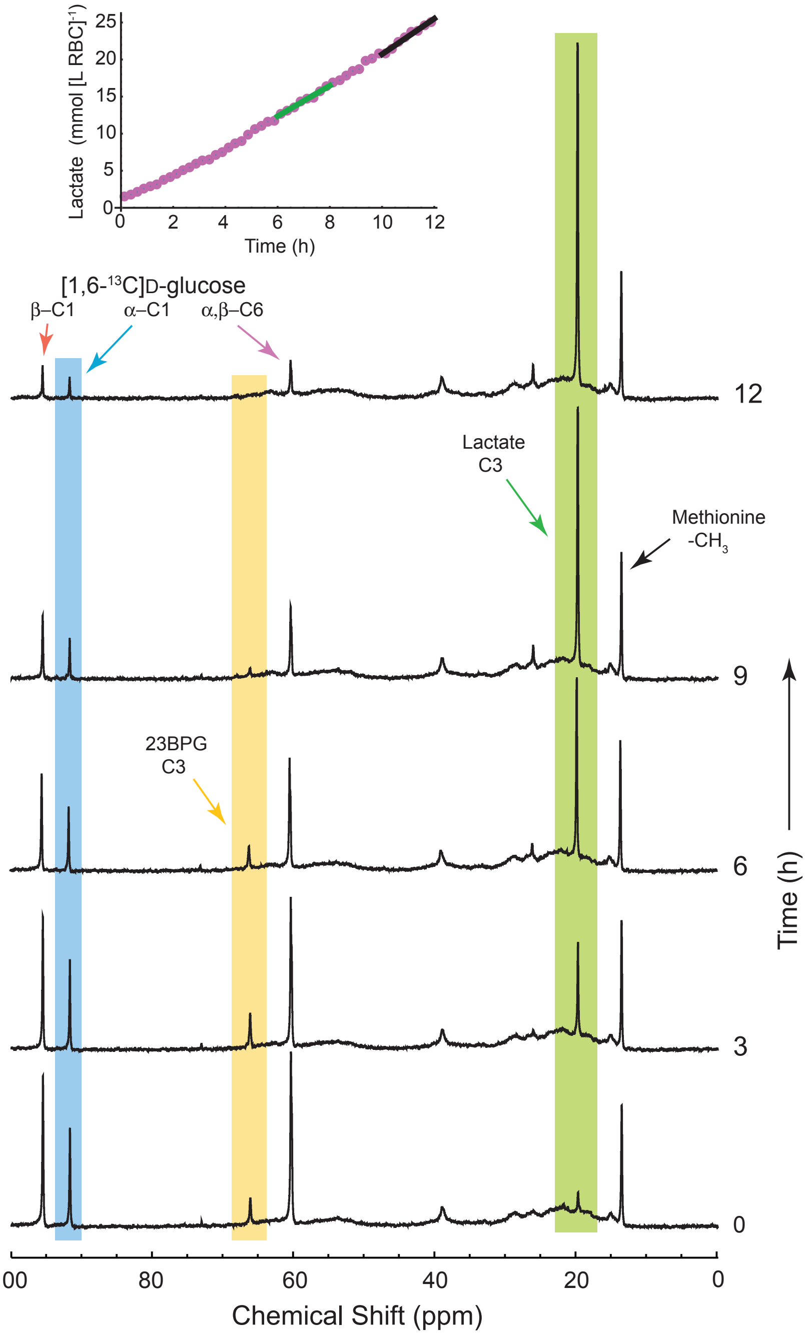

Figure S6. ^{13}C NMR (100.46 MHz) spectral time course showing glycolysis in RBCs (not loaded with 5FBAPTA) at ~34ºC. The control sample of 3 mL RBCs (*Ht* = 0.81) was supplemented with [1,6-^{13}C]D-glucose and $^{13}CH_3$-L-methionine (final concentrations averaged over the sample volume of 8 mM and 7 mM, respectively). Each spectrum was recorded over 15 min with only every 12th spectrum shown (centre-time of spectral acquisition indicated on the right). NMR settings were as for Fig. 5. The lactate resonance is highlighted

in green, and the inset shows the corresponding integrals (pink dots). The black line is from linear regression onto the last eight data points (10 – 12 h; (-[3.30 ± 1.4] + [2.40 ± 0.13] t) and the green line ([0.015 ± 0.85] + [2.06 ± 0.12] t) from eight points centred on 7 h. Yellow highlights the rise from an initial value and then the fall to 0 of 23BPG; and blue highlights the α-anomer of glucose that declined in parallel with the two other glucose peaks. The integral of the methionine resonance at 13.0 ppm remained constants within experimental error. The concentration of glucose, 23BPG and lactate were measured by importing the spectral data from TopSpin into a *Mathematica* program that performed automatic baseline correction and scaling to the known amount of $^{13}CH_3$-L-methionine (that is not metabolised by human RBCs).

The progress curve shows the initial transient stage that is always seen (e.g.,[5]) in such experiments. It is due to the washout of unlabelled intermediates in the pentose phosphate pathway, glycolysis, and 23BPG into ^{12}C-L-lactate. The rate of lactate production at ~7 h had stabilized to 2.06 mmol (L RBC)$^{-1}$ h^{-1} and was similar 5 h later at 2.40 ± 0.13 mmol (L RBC)$^{-1}$ h^{-1}. The time course was carried out at a setting of 30°C on the NMR-probe thermostat, but some RF heating was known to occur from trial experiments so the estimated sample temperature was ~34°C. The expected rate of lactate production under the same conditions of sample preparation is 3 mmol (L RBC)$^{-1}$ h^{-1} so the RBCs in the present study performed glycolysis normally. This was crucial to establish, because an additional feature of the preparation was inclusion of 10 mM dithioerythritol (DTE) and 1% BSA w/v in the suspension medium. The -SH reagent was included to reduce the possibility of echinocyte formation as we considered that this alone might contribute to a change in mean membrane curvature and hence stimulation of Piezo1, quite apart from mechanically induced distortion. Thus, DTE was included in the gel-RBC stretching experiments reported in Fig. 1.

Supplementary References

1. Robertson, W. G. & Marshall, R. W. Calcium measurements in serum and plasma – total and ionized. *CRC Crit. Rev. Clin. Lab. Sci.* **11**, 271-304 (1979).

2. Krause, K. H. *et al.* The calcium signal and neutrophil activation. *Clin. Biochem.* **23**(2), 159-166 (1990).

3. Deuticke, B. Chapter 2 Membrane lipids and proteins as a basis of red cell shape and its alterations. In *Red Cell Membrane Transport in Health and Disease* (eds Bernhardt, I. & Ellory, J. C.) 748 (Springer, Berlin, 2003).

4. Lew, V. L. & Tiffert, T. On the mechanism of human red blood cell longevity: roles of calcium, the sodium pump, PIEZO1, and Gárdos channels. *Front. Physiol.* **8**, 977 (2017).

5. Kuchel, P. W. & Shishmarev, D. Accelerating metabolism and transmembrane cation flux by distorting red blood cells. *Sci. Adv.* **3**(10), 1016 (2017).
6. Cöulfen, H. & Borchard, W. Determination of the partial specific volumes of thermoreversible gelatin/water and κ-carrageenan/water gels. *Macromol. Chem. Phys.* **195**, 1165-1175 (1994).
